# Characterizations of novel broad-spectrum lytic bacteriophages *Sfin-2* and *Sfin-6* infecting MDR *Shigella* spp. with their application on raw chicken to reduce the *Shigella* load

**DOI:** 10.3389/fmicb.2023.1240570

**Published:** 2023-11-29

**Authors:** S. K. Tousif Ahamed, Srijana Rai, Chiranjib Guin, Rameez Moidu Jameela, Somasri Dam, Dhiviya Prabaa Muthuirulandi Sethuvel, V. Balaji, Nabanita Giri

**Affiliations:** ^1^Department of Microbiology, Acharya Prafulla Chandra College, New Barrackpore, Kolkata, India; ^2^Department of Microbiology, Bose Institute, Kolkata, West Bengal, India; ^3^Department of Microbiology, The University of Burdwan, Bardhaman, West Bengal, India; ^4^Department of Research and Development, Bioberrys Healthcare and Research Centre, Vellore, Tamil Nadu, India; ^5^Department of Clinical Microbiology, Christian Medical College, Vellore, Tamil Nadu, India

**Keywords:** bacteriophage, *Shigella* spp., phage therapy, genome sequencing, large terminase

## Abstract

The evidence and prevalence of multidrug-resistant (MDR) *Shigella* spp. poses a serious global threat to public health and the economy. Food- or water-borne MDR *Shigella* spp. demands an alternate strategy to counteract this threat. In this regard, phage therapy has garnered great interest from medical practitioners and researchers as a potential way to combat MDR pathogens. In this observation, we isolated *Shigella* phages from environmental water samples and tested against various clinically isolated MDR *Shigella* spp. In this study, we have defined the isolation and detailed physical and genomic characterizations of two phages *Sfin-2* and *Sfin-6* from environmental water samples. The phages exhibited potent lytic activity against *Shigella flexneri, Shigella dysenteriae*, and *Shigella sonnei*. They showed absorption within 5–10 min, a burst size ranging from ~74 to 265 PFU/cell, and a latent period of 5–20 min. The phages were stable at a broad pH range and survived an hour at 50°C. The purified phages *Sfin-2* and *Sfin-6* belong to the *Siphoviridae* family with an isometric head (64.90 ± 2.04 nm and 62.42 ± 4.04 nm, respectively) and a non-contractile tail (145 ± 8.5 nm and 148.47 ± 14.5 nm, respectively). The *in silico* analysis concluded that the size of the genomic DNA of the *Sfin-2* phage is 50,390 bp with a GC content of 44.90%, while the genome size of the *Sfin-6* phage is 50,523 bp with a GC content of 48.30%. A total of 85 and 83 putative open reading frames (ORFs) were predicted in the *Sfin-2* and *Sfin-6* phages, respectively. Furthermore, a comparative genomic and phylogenetic analysis revealed that both phages represented different isolates and novel members of the T1-like phages. *Sfin-2* and *Sfin-6* phages, either individually or in a cocktail form, showed a significant reduction in the viable *Shigella* count on raw chicken samples after 72 h of incubation. Therefore, these results indicate that these phages might have a potential role in therapeutic approaches designed for shigellosis patients as well as in the biological control of MDR *Shigella* spp. in the poultry or food industry during the course of meat storage.

## 1 Introduction

Shigellosis or bacillary dysentery is an acute inflammatory diarrheal disease in most of the developing countries affecting nearly 165 million people each year (WHO, 2013). Though the number of reported deaths has decreased, shigellosis still causes high morbidity and mortality, particularly among children and young adults (Sur et al., 2004). The genus *Shigella* having four pathogenic serogroups (*Shigella dysenteriae, Shigella flexneri, Shigella boydii*, and *Shigella sonnei*) is mainly associated with Shigellosis (Kotloff et al., 1999; Yang et al., 2018). The main mode of transmission is via the fecal-oral route due to the intake of contaminated food and water (Baird-Parker, 1994; Shahin et al., 2019b; Pakbin et al., 2021). The World Health Organization (WHO) recommends antibiotics for the treatment of Shigellosis; nonetheless, the extensive use of antibiotics can lead to the development of multidrug-resistant (MDR) *Shigella* species (Sivapalasingam et al., 2006; von Seidlein et al., 2006; Muthuirulandi Sethuvel et al., 2017; Puzari et al., 2018). Although, recently, there have been some antibiotics suggested for the treatment, including ciprofoxacin [a fuoroquinolone (FQ)], pivmecillinam, azithromycin, and ceftriaxone (a third-generation cephalosporin) (Nandy et al., 2010; Tariq et al., 2012; Azmi et al., 2014), drug-tolerant persister *S*. *flexneri* and FQ-resistant *Shigella* spp. have still been identified in many Asian countries, including India (Taneja and Mewara, 2016; Puzari et al., 2018; Sethuvel et al., 2019). Hence, the repetitive transition in the antimicrobial resistance behavior of *Shigella* hinders the development of standard drugs against shigellosis. The potential ability of these bacteria to gain and disperse exogenous genes through mobile genetic elements, such as plasmids, transposons, insertion sequences, and genomic islands, is mainly responsible for the emergence of their multidrug-resistant strains (Muthuirulandi Sethuvel et al., 2019; Ranjbar and Farahani, 2019).

Bacteriophages are specific viruses that have the capability to infect and kill their target bacterial cells (Ayariga et al., 2018; Li et al., 2020; Ayariga Joseph et al., 2021; Gildea et al., 2022a,b; Ibrahim et al., 2023). The characteristics of bacteriophages, such as ubiquitous nature, host specificity, safety, antimicrobial activity, and surface decontamination ability, make them a suitable agent for therapeutic purposes (Peng et al., 2019). Currently, antibiotic resistance is a difficult problem to overcome, and due to the host specificity, phages are not ideal for broad-spectrum use; however, a combination of different bacteriophages, known as phage cocktail, can be an ideal means to combat antibiotic-resistant bacterial strains, since the bacterial cocktail increases the host range of the phages (Lin et al., 2017).

There have been recent reports of several bacteriophages against *Shigella* spp. The lytic *Shigella* phages vB_SflS-ISF001, vB_SsoS-ISF002, and pSf-1 infect both *S. flexneri* and *S. sonnei* (Jun et al., 2013; Shahin et al., 2018, 2019a). The lytic phage Sfk20 infects *S. flexneri* serotypes1b, 2a, 3a, *S. sonnei*, and *S. dysenteriae* 1 but is ineffective against *S. flexneri* serotypes *4, 6*, and *S. boydii* (Mallick et al., 2021). The novel lytic phage *Sfin-1* infects MDR *S. flexneri, S. dysenteriae*, and *S. sonnei* along with *Escherichia coli* C (Ahamed et al., 2019). The microviridae phage SGF3 has been reported to infect *S. flexneri* (Lu et al., 2022). Moreover, a number of phages that are involved in the serotype conversion of *S. flexneri* have been discovered, which includes SfII, Sf6, SfV, and SfX (Allison and Verma, 2000).

In addition to water, food can also serve as a possible indirect mode of transmission of *Shigella*. There are reports of the isolation of *Shigella* from different foods, including fresh vegetables, cooked chicken meat, salads, fruits, and dairy products, ultimately leading to Shigellosis outbreaks (Shahin and Bouzari, 2018; Pakbin et al., 2022). Globally, foodborne *Shigella* is estimated to cause 1–3 million disability-adjusted life years (DALYs) (Havelaar et al., 2015). Moreover, a recent analysis of *Shigella* isolates from more than 1,600 food samples, such as seafoods, fresh vegetables, and meats, revealed that 89% of the isolated *Shigella* strains were multidrug-resistant (Marami et al., 2018; Pakbin et al., 2021). Hence, effective measures are necessary to reduce *Shigella*-associated food-borne outbreaks and prevent the spread of resistant bacteria. One potential solution to this issue is the use of bacteriophages. In addition, using a mixture of several different phages, i.e., phage cocktail, provides a highly collaborative effect for antibacterial strength and a broad host range compared to using a single phage (Chan et al., 2018; Costa et al., 2019; Shahin et al., 2021).

Thus, the current study reports the isolation and detailed physical and genomic characterizations of two novel lytic bacteriophages *Sfin-2* and *Sfin-6*. In addition, the efficacy of the novel bacteriophage cocktail consisting of these two *Shigella* phages was investigated based on their ability to reduce *Shigella* loads on raw chicken ready-to-eat meat.

## 2 Materials and methods

### 2.1 Bacterial strains and multidrug resistance test

The study analyzed 50 MDR clinical isolates of *S. flexneri, S. dysenteriae, S. sonnei, S. boydii*, and *Salmonella enterica* serovar Typhi, as well as various strains of *E. coli* such as AG100, K12, XL1 Blue, and *E. coli* C. The stool samples of patients were collected at the Bacteriology Division of National Institute of Cholera and Enteric Diseases (NICED), Kolkata and Christian Medical College (CMC), Vellore, India to obtain all *Shigella* and *Salmonella* strains, which have been reported earlier (Table 1) (Muthuirulandi Sethuvel et al., 2017; Ahamed et al., 2019; Sethuvel et al., 2019). For the purpose of conducting various experiments, the Luria broth (LB) with or without antibiotics was used for growing bacterial strains at 37°C. Then, the growth of these strains was checked by measuring the absorbance at 600 nm.

**Table 1:** Host specificity test for several clinically isolated MDR strains to *Sfin-2* and *Sfin*-6 phages isolated from the water samples of Ganga river in Kolkata, West Bengal, India.

**Sl. No**.	**Strain ID**	**Bacterial isolates with different serotypes**	**Antimicrobial resistance profile by disc diffusion method**	**Lysis by Sfin-2**	**Lysis by Sfin-6**
1.	BCH5722	*Shigella flexneri 2a (1A)*	ACTQNaCipNorOfx	**+**	**+**
2.	BCH4025	*Shigella flexneri 2a (2A)*	ACQ	**+**	**+**
3.	BCH3651	*Shigella flexneri 2a (3A)*	ACTQ	**+**	**+**
4	BCH3557	*Shigella flexneri 2a (4A)*	CTQNa	**+**	**+**
5	BCH7151	*Shigella flexneri 2a (5A)*	ACTQNaCipNorOfx	**+**	**+**
6	CMCFC2181	*Shigella flexneri (1)*	AQCipCefSxt	**+**	**+**
7	BCH5762	*Shigella dysenteriae 1(1A)*	ACTQNaCipNor	**+**	**+**
8	BCH5848	*Shigella dysenteriae 1(2A)*	ACTQNaCipN	**+**	**+**
9	BCH5859	*Shigella dysenteriae 1(3A)*	ACTQNaCipNorOfxAzm	**+**	**+**
10	BCH5912	*Shigella dysenteriae 1(4A)*	ACTQNaCipNorOfx	**+**	**+**
11	BCH5946	*Shigella dysenteriae 1(5A)*	ACTQNaCipNorOfxCef	**+**	**+**
12	CMCFC2358	*Shigella dysenteriae (1)*	AQCipCef	**+**	**+**
13	BCH7084	*Shigella sonnei (1)*	TQNa	**+**	**+**
14	BCH7264	*Shigella sonnei (2)*	TQNa	**+**	**+**
15	CMCFC87	*Shigella sonnei*	AQNaCipCefSxt	**+**	**+**
16	CMCFC1799	*Shigella sonnei*	AQNaCipCefSxt	**+**	**+**
17	BCH3143	*Shigella boydii (1)*	TQNa	**–**	**–**
18	BCH4324	*Shigella boydii (2)*	TQNa	**–**	**–**
19	CMCFC2293	*Shigella boydii*	AQNaCipCefSxt	**–**	**–**
20	BCR62	*Salmonella enterica serovar* Typhi *(1)*	NaCipNorAzm	**–**	**–**
21	BCR43	*Salmonella enterica serovar* Typhi (2)	NaAzm	**–**	**–**
22		*Escherichia coli* K12		**–**	**–**
23		*Escherichia coli* C		**–**	**–**
24		*Escherichia coli* AG100		**–**	**–**
25		XL1 Blue		**–**	**–**

BCH, Bidhannagar Children Hospital; BCR, Bidhan Chandra Roy Hospital; CMC, Christian Medical College, Vellore; A, ampicillin; C, chloramphenicol; T, tetracycline; Q, cotrimoxazole; Na, nalidixic acid; Cip, ciprofloxacin; Nor, norfloxacin; Ofx, ofloxacin; Azm, azithromycin; Cef, cefixime; Sxt, sulfamethxazol.

### 2.2 Isolation, amplification, and purification of bacteriophages

Environmental water samples were collected from the Ganga river, near Barrackpore, North 24 Parganas district and Sreerampore, Hoogly district, which is ~25 km from Kolkata, West Bengal, India. The collected water samples were then filtered through filter paper (Whatman 1) to remove the particulate matter. The log phase *S. flexneri* 2a culture was added to the water sample with 10% (w/v) peptone following the incubation at 37°C for 24 h with shaking. To remove the bacterial debris, 1% (w/v) chloroform was mixed with the culture and then shaken properly. Furthermore, after the centrifugation of the mixture, the supernatant was collected and filtered through a 0.22-μm pore membrane (Millipore, USA). A volume of 10 μl of the filtrate was inoculated as a spot on a *Shigella* spp. plate, with subsequent formation of a clear zone around the spot indicating the presence of the bacteriophage against *S. flexneri* 2a. In addition, the other *Shigella* spp. serotypes were also included in the study.

The water samples were then used for plaque assay; 200 μl *Shigella* culture (OD600 = 0.3) and 100 μl filtrate were mixed together with 3.5 ml soft agar (0.9%), and finally, LB hard agar plate was used for plating. After the incubation of the plate at 37°C for 24 h, clear distinct plaques developed on the plate, which was then transferred to a separate *Shigella* plate. An individual plaque was shifted into a 500-μl phage dilution medium (0.85% sodium chloride and 0.1% tryptone). An additional round of plaque assay was done using the above suspended phage solution. In this way, each plaque was transferred three times for the purification of the bacteriophage.

Further, the dilutions and assaying of the phage were done to obtain a confluent lysis plate. The scrapping of the layer of soft agar was carried out and dissolved in a cold phage dilution medium (0.85% sodium chloride, 0.1% tryptone), which was retained on ice for 24 h. The supernatant was then collected after centrifugation at 5,000 × g, and the phage lysate obtained was pelleted at 68,000 g for 2 h at 4°C in an ultracentrifuge, which resulted in a higher phage titer value. Moreover, cesium chloride (CsCl) density gradient centrifugation was performed (ρ = 1.3, 1.5, 1.7 g/ml) at 100,000 g for 3 h at 4°C to obtain increased purification. The phage band captured between 1.7 and 1.5g/ml was gathered and then dialyzed against Tris-HCl magnesium sulfate (TM) buffer (50 mM Tris-Cl, pH 8.0 with 10 mM MgSO_4_). Finally, the phage was stored at 4°C.

### 2.3 Host range determination

The different strains of *Shigella, Salmonella*, and *E. coli* were used for determining the host range of isolated phages (Table 1). After growing them through the night in nutrient broth at 37°C, 200 μl of the bacterial cell culture was mixed with 3.5 ml of the molten soft agar (0.7% w/v) and overspread onto the surface of solid basal LB agar (1.5% w/v). A suspension phage of 10 μl (about 1.0 × 10^10^ PFU/ml) was used for spotting onto the bacterial lawn, which was then incubated overnight at 37°C. Clear lysis of the spot where the phage suspension was inoculated indicated the sensitivity of the bacteria. Each test was repeated three times. There were two categories of spots according to the degree of clarity: clear (+) and no reaction (–).

### 2.4 Thermal and pH stability

The thermal stability testing was performed using 1 ml of phage particles (~16 × 10^13^ pfu/ml for *Sfin-2* and 15 × 10^15^ pfu/ml for *Sfin-6)*, which were incubated at 4, 37, 50, 60, 70, 80, and 90°C, with aliquots (100 μl) taken for each temperature after 5, 15, 40, and 60 min and titered by the double-layered plaque assay against *Shigella* spp. Similarly, the pH stability testing was performed on phage particles (about 16 × 10^12^ pfu/ml*)* that were placed in 1 ml of TM buffer at different pH ranges between 2 and 12 (modified using HCl or NaOH for acidic or alkaline range, respectively) for 1 h at 37°C. The aliquots (100 μl) from each pH were then titered by the double-layered plaque assay against *Shigella* spp. (Wei et al., 2021).

### 2.5 Transmission electron microscopy

Ultrapure phages obtained from CsCl purification were used for electron microscopic imaging. The imaging was done at the Electron Microscopy Laboratory, University of Burdwan, West Bengal. The bacteriophage suspension (~1 × 10^22^ pfu/ml) was transferred onto the grid using a Gilson pipette and negatively stained with a 2% (w/v) uranyl-acetate solution. Then, it was examined under a JEOL JEM-1400Plus transmission electron microscope with an operating voltage of 200 kV.

### 2.6 Lytic activity of *Sfin-2* and *Sfin-6*

According to the CLSI guidelines, the isolated *Shigella* strains were extensively drug-resistant (CLSI, 2021). The method described by Wang et al. (2016) with some modifications was used for determining the bacteriolytic activity of phages. In the presence of various antibiotics, such as ampicillin (32 μg/ml), chloramphenicol (32 μg/ml), tetracycline (16 μg/ml), cotrimoxazole (25 μg/ml), nalidixic acid (32 μg/ml), ciprofloxacin (4 μg/ml), norfloxacin (16 μg/ml), and ofloxacin (8 μg/ml), the cells of *S. flexneri* 2a (strain IDBCH5722, Table 1) and *S. dysenteriae* 1 (strain IDBCH5762, Table 1) were grown. Similarly, in the presence of tetracycline (16 μg/ml), cotrimoxazole (25 μg/ml), and nalidixic acid (32 μg/ml), *S. sonnei* (strain IDBCH7084, Table 1) was grown. After centrifugation, 20 ml of cultures (OD600 = 0.3) were resuspended in 1 ml of freshly prepared LB. Furthermore, after adding phages at different multiplicity of infection (MOI) of 0.1, 0.01, and 0.001, they were allowed to adsorp for 5 min (*S. flexneri* 2a and *S. dysenteriae* 1) or 10 min (*S. sonnei* 1) at 37°C. Thereafter, the individual suspension was transferred to 20 ml of freshly made LB. At specific time intervals of 5 h duration, aliquots were taken and the bacterial cell count was recorded using the spread plate technique. The bacterial cultures inoculated only with phage dilution medium and respective antibiotics were used as the negative control.

### 2.7 One-step growth curve

A one-step growth curve experiments was executed by a procedure stated by Malek et al. (2009) with an alteration. Concisely, *Shigella* spp. (*S. flexneri* 2a, *S. dysenteriae* 1, and *S. sonnei* 1) were cultured in LB medium at 37°C with respective antibiotics. After centrifuging 20 ml of *Shigella* culture (OD600 = 0.3) at 5,000 × g at 4°C for 10 min, the resulting pellet was resublimed in 1 ml of fresh LB. Then, the phage particles at an MOI of 0.01 were mixed with *Shigella* culture. Thereafter, the suspension was incubated for enhanced adsorption (5 min for *S. flexneri* 2a and *S. dysenteriae* 1, 7 min for *S. sonnei* 1) at 37°C pursued by 10^4^-fold of dilution with 10 ml as final volume. Subsequently, during the incubation process at 37°C, 100 μl of aliquots were taken at different time intervals up to 100 min. These samples were then mixed with 200 μl of *Shigella* culture, and a double-layered agar plate assay to determine the phage titration was performed. The above experiments were carried out three times for each *Shigella* spp. The determination of the burst size was calculated as a ratio of the average bacteriophage particles produced after the burst and the average number of phage particles adsorbed.

### 2.8 Genome sequencing and analysis

The phage samples were allowed for ultra-purification just before DNA extraction as described. A sterile 2 ml centrifuge tube (Tarsons, India) was filled with 450 μl of phage lysate. After adding 1 μl of DNase I (2,000 units/ml, NEB, USA) and 5 μl of RNaseA (10 mg/ml, Thermo Scientific, USA) to the solution, it was incubated at 37°C for 1 h. Each centrifuge tube was treated with 5 mM EDTA and then incubated at 78–80°C for 20 min to denature DNase I. Then, 250 μg of Proteinase K (SRL, Mumbai, India) was added with incubation for 2 h at 55°C. After the primary treatment of the phage sample, the genomic DNA was isolated using the phage DNA isolation kit (Norgen “Canada”) as per the manufacturer's instruction with modifications (Berg et al., 2016).

The kit ION Xpress (S5-00205) version 5.0.4. was utilized for accomplishing whole genome sequencing of phages. The quality of the sequence data was checked using PRINSEQ, and the reads were quality-trimmed/filtered. The filtered sequence was converted into a single contig using SPAdes 3.8.0 (Bankevich et al., 2012). Rapid Annotation Subsystem Technology (RAST) was used for the accomplishment of genome annotation (Aziz et al., 2008). The resulting nucleotide sequence of the phage genome was submitted at GenBank under accession numbers MK972831 (*Sfin-2*) and MN393473 (*Sfin-6*), respectively. By using the BLASTp program and conserved domain search (http://www.ncbi.nlm.nih.gov/), the function of the proteins encoded by various coding sequences (CDSs) was speculated (Table 2). The possible origin of replication was predicted by GeneSkew program (http://genskew.csb.univie.ac.at/). The Neural Network Promoter Prediction tool of the Berkeley Drosophila Genome Project was used to predict putative promoter regions (minimum promoter score: 0.9, http://www.fritfly.org/seq_tools/promoter.html). The ARNOLD terminator finding program was used for determining Rho-independent transcription terminators (Lesnik et al., 2001). The tRNA scan-SE search program (http://lowelab.ucsc.edu/tRNAscan-SE/) was used for identifying putative tRNAs, if any of them was present (Lowe and Chan, 2016). The Mauve procedure was conducted for whole genome comparisons (http://asap.ahabs.wisc.edu/mauve/).

**Table 2:** Characteristics of the protein coding sequences of phage *Sfin-2* and *Sfin-6* according to the homology to protein database.

**Predicted functional CDSs**	**Best blastp match and identity (%) and protein family**	**CDS**	**Start**	**Stop**	**Length (bp)**	**CDS**	**Start**	**Stop**	**Length (bp)**

		***Sfin-2***				***Sfin-6***			
Tail fiber protein	*Sfin-1*, 100% pfam09327COG4733	1	3,807	358	3,450	8	10,302	6,853	3,450
Tail fiber	*Sfin-1*100%	5	6,425	6,072	354	2	1,050	2,834	1,784
Tail fiber	Escherichia phage vB_EcoS_Chao	10	10,756	10,088	669	12	12,920	12,567	354
Tail fiber	*Sfin-1* 98%	78	44,946	45,809	864	16	17,252	16,584	669
Tail assembly protein	*Sfin-1*, 100 % cl01945	2	4,483	3,884	600	9	10,978	10,379	600
Tail assembly protein	*Sfin-1*, 100 % cd08073	3	5,214	4,480	735	10	11,709	10,975	735
Minor tail protein	phi2457T, 100 % cl01908	4	5,993	5,211	783	11	12,488	11,706	783
Minor tail protein	*Sfin-1*, 100 % cl01940	79	45,923	46,729	807				
Tail length tape-measure protein	phi2457T, 100 % COG4942	6	7,871	6,492	1,380	13	15,795	12,988	2,808
Tail length tape-measure protein	phi2457T, 100 % pfam06791	7	9,298	7,916	1,383				
Capsid and scaffold protein	phi2457T, 100 % COG2369	19	16,224	15,112	1,113	25	22,721	21,609	1,113
Minor capsid protein	Shigella phage phi2457T, 100%	20	16,988	16,227	762	26	23,485	22,724	762
Terminase large subunit	*Sfin-1*, 100 % COG5410	22	19,886	18,318	1,569	28	26,383	24,815	1,569
Terminase small subunit	Shfl1, 100 % pfam16677	23	20,450	19,926	525	29	26,947	26,423	525
3′-phosphatase, 5′-polynucleotide kinase	Sfin-3, 97%	32	23,523	22,990	534	38	30,021	29,488	534
Holin protein	Echerichia phageADB-2, 100%	65	37,499	37,284	216	72	43,999	43,784	216
DNA adenine methyltransferase	ADB-2, 100 % cl05442	72	40,805	40,092	714	79	47,306	46,593	714
DNA helicase	*Sfin-1*, 100 % COG1061	74	43,076	41,286	1,791	81	49,576	47,786	1,791
DNA helicase	Escherichia phage ADB-2, 95% cl28899	75	43,303	43,067	237	82	49,803	49,567	237
DNA primase	VbEcoS SA12KD, 99% smart00778	77	43,925	44,845	921	1	30	992	963
Recombinase	Escherichia phage vB_EcoS_SA30RD, 99% pfam04404	81	47,865	47,218	648	4	3,970	3,323	648
Exonuclease	*Sfin-1*, 100% cl00641	82	49,004	47,940	1,065	5	5,109	4,045	1,065

### 2.9 Genome end determination of isolated phages

A comparative analysis of the phylogenetic relationships between amino acid sequences of phage terminase large subunit and those of the other phages of a familiar packaging system can be performed for recognizing the procedure of phage packaging and determining the bacteriophage genome ends (Amarillas et al., 2017). Hence, the recreation of the phylogenetic tree was done using the phages with the amino acid sequences of the large terminase. In addition, the relationships between *Sfin-2* and *Sfin-6* phages and the other phages were analyzed. For accomplishing the phylogenetic analysis, the predicted amino acid sequences of the large terminase subunit genes of the phages were retrieved from National Center for Biotechnology Information (NCBI). In this study, molecularly analyzed bacteriophages are implicated containing well-characterized dsDNA, which has different types of packaging strategies that are dependent on terminase actions (headful, 5′-extended cos ends, 3′-extended cos ends, and direct terminal repeats). ClustalW in MEGAX with default parameters were used for aligning all the sequences. The neighbor-joining method was used to construct a phylogenetic tree, and phylogenies were determined by the bootstrap value of 1,000 replicates in MEGA X.0 version (Filipski et al., 2014). Furthermore, the genome ends were recognized as shown by Amarillas and Leon-Felix (Amarillas et al., 2017). Approximately 1 μg bacteriophage DNA was digested with separate restriction enzymes (BglII, MluI) as per the manufacturer's guidelines (NEB, USA) for identifying the presence of terminally redundant genome ends that were circularly permutated. The digests produced were then heated to 80°C for 15 min followed by cooling quickly in ice or slowly at ambient temperature. Then, the digests were loaded and run on agarose gel (0.8% w/v) in TAE electrophoresis buffer after which the gel was stained with ethidium bromide (EtBr) and visualized with UV illumination. Lastly, as a DNA molecular weight marker, GeneRuler 1 kb Plus DNA Ladder (Thermo Fisher Scientific, USA) was used.

### 2.10 Characterization of the phage receptor

To determine the receptor features of *Sfin-2* and *Sfin-6* for phage host interaction, the following experiments were performed as described earlier with certain alterations (Kiljunen et al., 2011). To determine the proteinase K effect on the adsorption of phages*, S. flexneri* 2a (OD600 = 0.3) was used. The host was subjected to proteinase K treatment (250 mg/ml, SRL, Mumbai, India) for 2 h at 55°C and was left for adsorption analysis at an MOI of 0.0001. Furthermore, *S. flexneri* 2a cells were centrifuged at 5,000 × g for 5 min to determine the inhibitory action of periodate on the phage–host interaction. The pellets so obtained were dissolved into 50 mM sodium acetate (pH 5.2) solution in the presence or absence of 200 mM NaIO_4_ and then incubated for 2 h in the dark. An adsorption assay was carried out with the washed cells following the incubation. Again, for *Sfin-2*, the *S. flexneri* 2a cell was primarily treated with proteinase K and allowed for a secondary treatment with periodate. Moreover, without proteinase K and sodium acetate, a control experiment was also performed to confirm that the probable effect is not the result of sodium acetate and host cell incubation at 55°C. For both of these assays, as a non-absorbing control, LB medium was used. In the control supernatant, the phage titer value was adjusted to 100%.

### 2.11 Efficacy of the isolated phages to reduce the *S. flexneri* 2a load on raw chicken samples by a single phage and cocktail phages

In the area where the present study was carried out, chicken is considered as a primary meat source among the meat-based food, thereby increasing the risk of *Shigella* spp contamination. Raw chicken was used in this experiment (Shahin and Bouzari, 2018). The chicken was collected from a local shop and sliced aseptically under a biosafety cabinet. The pieces were then placed on sterile petridishes and stored at 4°C until further use. *Shigella flexneri* 2a was grown in antibiotics containing LB broth at 37°C. Aseptically *S. flexneri* 2a cells (±10^9^ cfu) were carefully spread on the surface of the chicken pieces. The phage suspension of a single or cocktail of the two phages were put on to the surface of the inoculated chicken piece at a MOI of 0.1, followed by adsorption at room temperature for 10 min. Phage cocktail was prepared by adding an equal ratio of each phage. As a control, only phage suspension medium was used. After that, the treated and control samples were incubated at 4°C up to 96 h (Zhang et al., 2013). The number of viable *S. flexneri* 2a cells and the number of phages were measured at 0, 2, 24, 48, 72, and 96 h.

At each sampling time, the pieces of chicken were transferred to a sterile tube containing 5 ml of sodium magnesium (SM) buffer solution or 0.85% NaCl. Then, the samples were shaken at ambient temperature for half an hour. In order to harvest, the suspensions after transfer were centrifuged at 5,000 g for 10 min at room temperature. For phage-treated samples, the supernatant was collected in another microcentrifuge tube to ascertain the number of phages. In case of only host control, the pellet was washed thrice and resuspended in an equal volume of 0.85% NaCl solution. The bacterial cells were measured on HEA or XLD agar by the spread plate method, and the phage number was measured by plaque assay as mentioned previously.

### 2.12 Statistical analysis

To test the thermal stability, the titer value difference taken between 0 and 60 min were estimated for individual temperature. Student's *t*-test was applied for comparing the difference in the titer value for individual temperature to 4°C. To evaluate the details of bactericidal activity, two-way ANOVA test was performed. To analyze the phage receptor on the host cells, student's *t*-test was performed. To perform all statistical analysis, software GraphPad Prism 7.0 was used.

## 3 Results and discussion

### 3.1 Isolation of bacteriophages

**Figure 1:**
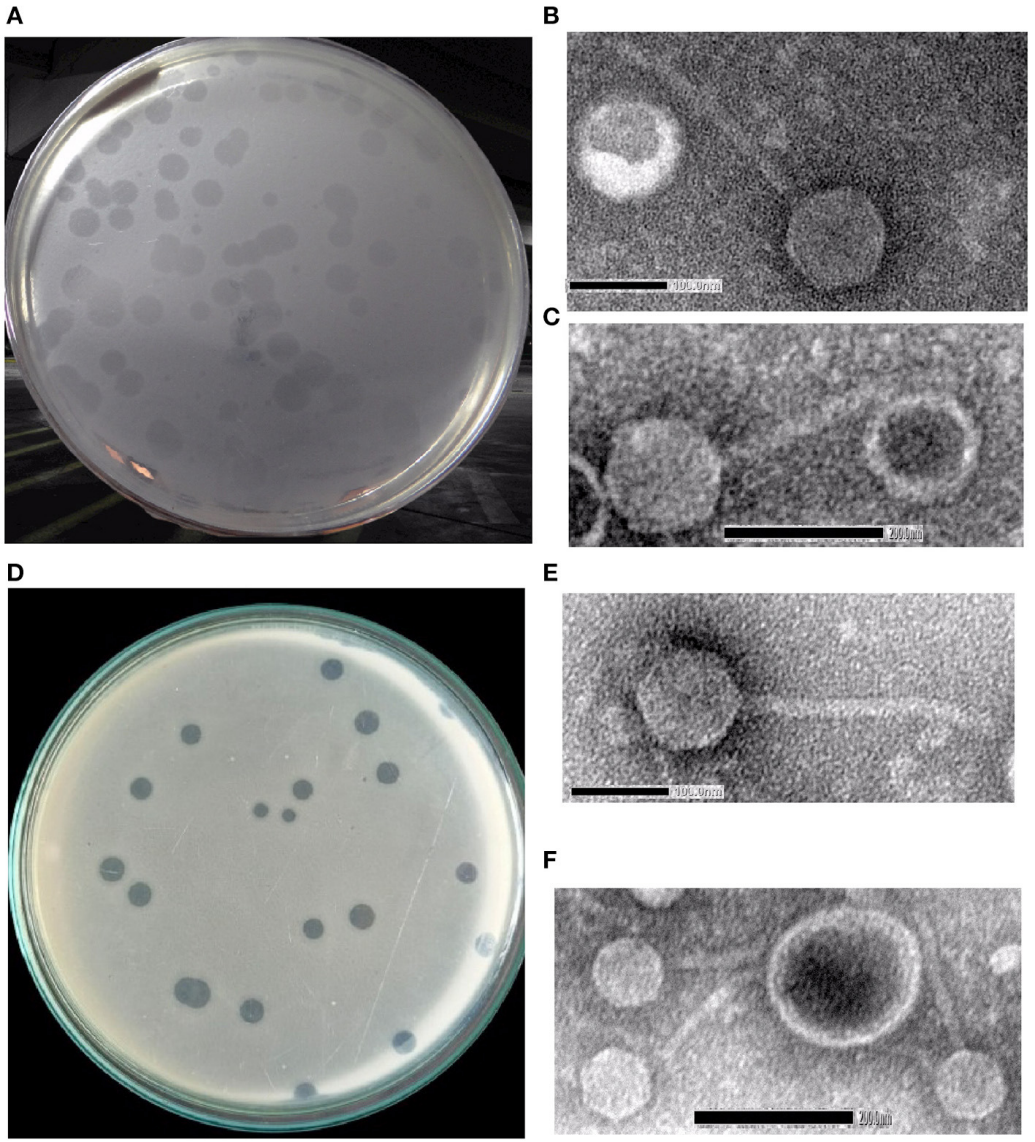
*Shigella* spp.-specific phages *Sfin-2* and *Sfin-6*. **(A, D)** Plaques of *Sfin-2* and *Sfin-6* in the lawn of *Shigella* spp. Ultra-purified phages were negatively stained and examined under electron microscope as described in Section 2. **(B, C, E, F)** The electronmicrograph broad view of the phages in 100 and 200 nm scales.

The water samples from River Ganga were collected from different regions in and around Kolkata, and *Shigella*-specific phages were determined by the methods as described in Section 2. Two phages named *Sfin-2* and *Sfin-6* were isolated from the waters of the River Ganga that could proliferate in various strains of clinically isolated MDR *Shigella* spp., and they formed clear plaques of size ranging from 1.3 to 1.9 mm in diameter with well-defined boundaries in the bacterial lawn after overnight incubation at 37°C (Figures 1A, D). The absence of more than one gene of specific phage proteins such as tail tape measure protein and large terminase subunit suggests the presence of a single type of phage in the sample.

### 3.2 Phage morphology

The morphology of purified *Sfin-2* and *Sfin-6* phages were observed using transmission electron microscopy (TEM), which revealed that *Sfin-2* and *Sfin-6* phages had an isometric head (64.90 ± 2.04 nm and 62.42 ± 4.04 nm, respectively) and a non-contractile tail (145 ± 8.5 nm and 148.47 ± 14.5 nm, respectively) anchored with a basal tuft (Figures 1B, C, E, F). The mature phage lacks a neck, base plate, spikes, or fiber. The structure of the phages according to the guidelines of the International Committee on Taxonomy of Viruses (ICTV) suggested that both of them belong to the family *Siphoviridae* and grouped into *Caudovirales* (Fauquet and Fargette, 2005).

The vast majority (over 95%) of the reported phages belong to the order *Caudovirales*, which are tailed phages. According to the Ackermann (1998), ~60% of the phages are classified under the family *Siphoviridae*, which have flexible and long tails.

### 3.3 Phage host range

Lytic spectrum of *Sfin-2* and *Sfin-6* phages were determined by spot test of pure phages on the lawn of different clinically isolated *S*. *flexneri, S*. *dysenteriae, S*. *boydii*, and *S. sonnei* with other enteropathogens such as *Salmonella typhi* and various *E. coli* strains, including XL1 Blue, AG100, K12, and *E. coli* C. The *Shigella* strains used in this study were resistant to various antibiotics such as amoxicillin, tetracycline, chloramphenicol, norfloxacin, ciprofloxacin, nalidixic acid, ofloxacin, cotrimoxazole, and azithromycin, which are frequently used for therapeutic purposes (Amezquita-Lopez et al., 2014) (Table 1). Spot tests revealed that both the phage suspensions, *Sfin-2* and *Sfin-6*, produced clear zones of inhibition against various serotypes of *S. flexneri, S. dysenteriae*, and *S. sonnei* but did not show activity against other bacterial species. This phenomenon clearly indicated that phages are polyvalent in nature.

While phages are usually very much specific, infecting only one species of bacteria, there has been a report of some polyvalent phages (Hamdi et al., 2017; Ahamed et al., 2019). The ability to lyse multiple *Shigella* strains highlighted that these phages could be explored for phage therapies against shigellosis. The wide host range of both the phages determined that the CDSs that encode host specific protein and tail component would be valuable. The main mode of transmission of *Shigella* spp. to humans is through the fecal-oral route; hence, the isolation of *Sfin-2* and *Sfin-6* phages indicated fecal contamination of the river.

### 3.4 *In vitro* bacterial challenge test

**Figure 2:**
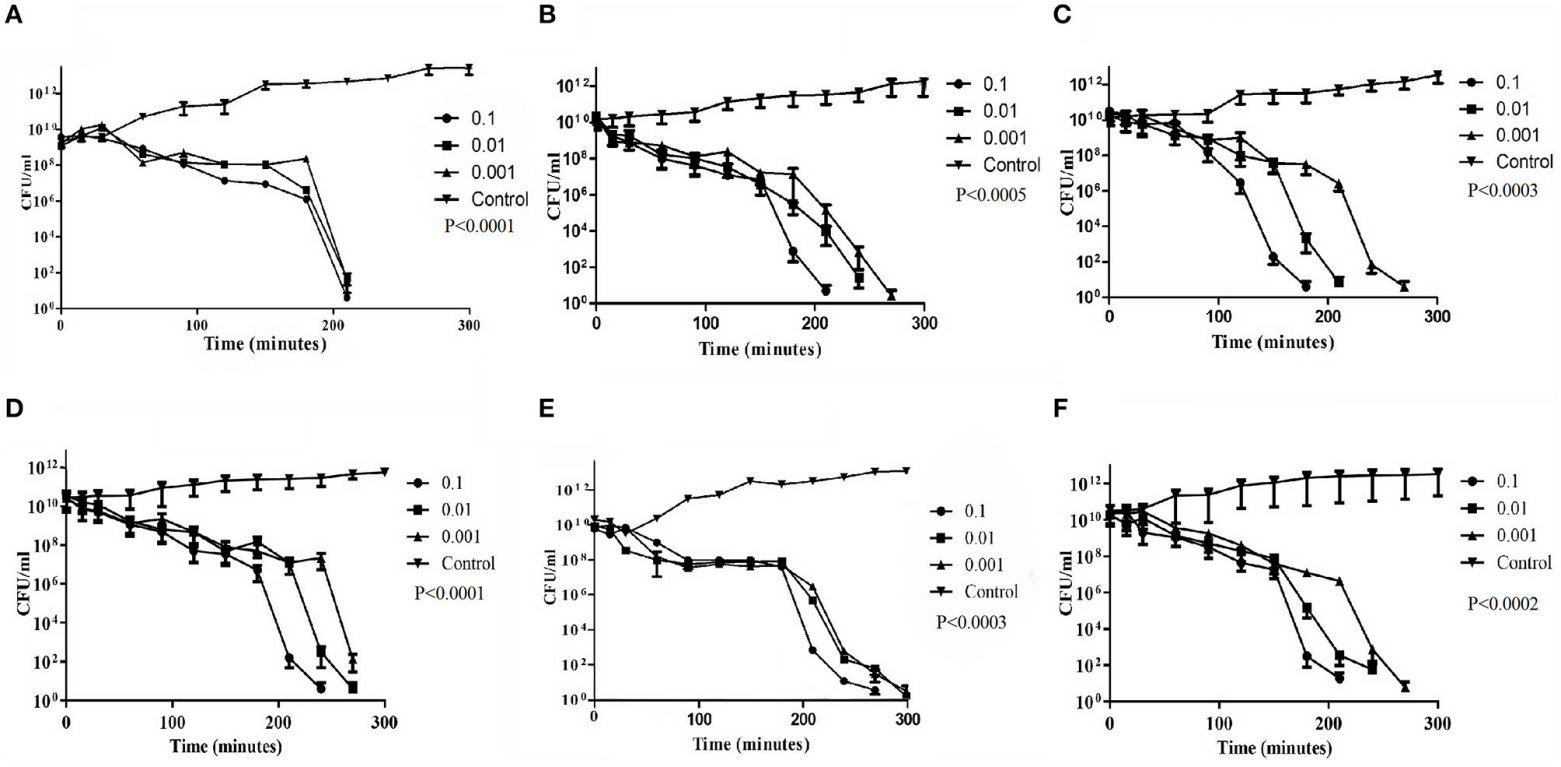
Bacterial challenge test of phage *Sfin-2* and *Sfin*-6 on different clinical isolates of *Shigella* spp. Clinically isolated species of **(A, D)** *Shigella flexneri* 2a **(B, E)** *Shigella dysenteriae* 1 and **(C, F)** *Shigella sonnei* 1 were grown (OD600 = 0.3) in 20 ml of LB medium in the presence of several antibiotics. They were harvested by centrifugation, resuspended in 1 ml of LB medium, and infected with both phages at MOIs of 0.1, 0.01, and 0.001. After adsorption, the cultures were diluted 21-fold in LB medium and incubated for 5 h with shaking at 37°C. At different time intervals, viability of *Shigella* spp. was determined by the spread plate method. As the negative control, *Shigella* spp. were grown only in the presence of antibiotics. The two-way ANOVA indicated significant difference between control and phage-infected sets (*p* < 0.0001, *n* = 3).

*In vitro* bacterial challenge tests were performed using both the phages, *Sfin-2* and *Sfin-6*, individually by adding the phage at an MOI of 0.1, 0.01, and 0.001 to mid-exponential phase cells (OD600 = 0.3) in the presence of multiple antibiotics chloramphenicol, ampicillin, tetracyclin, ciprofloxacin, cotrimoxazole, norfloxacin, and ofloxacin. For every single experiment, host strains were grown in the presence of respective antibiotics, whereas phage suspension medium was taken as control. Killing curves were generated by counting the viable colonies. For *Sfin-2*, the viability of bacterial cells was significantly decreased when infected with an MOI of 0.1, 0.01, and 0.001 and complete lysis occurred within 3.5 h in the case of *S. flexneri* 2a cells. For *S. dysenteriae*1, complete lysis occurred after 3.5 h of infection at an MOI of 0.1, while almost complete lysis occurred after 4.5 h at an MOI of 0.01 and 0.001. *Shigella sonnei* 1 cells were also significantly decreased, and complete lysis occurred after 3 h at an MOI of 0.1, while in the case of MOIs of 0.01 and 0.001, complete lysis occured after 3.5 h and 4.5 h of infections, respectively (*p* < 0.005; Figures 2A–C). In the case of *S. flexneri* 2a, complete lysis occurred after 3.5 h at an MOI of 0.1 and 4.5 h at MOIs of 0.01 and 0.001. The viability of bacterial cells were moderately decreased when *S. dysenteriae* 1 was infected with the phage *Sfin-6* at different MOI. Complete lysis occurred within 4.5 h at an MOI of 0.1, whereas complete lysis occurred at 5 h at MOIs of 0.01 and 0.001. The viable count of *S. sonnei* 1 cells were also decreased at an MOI of 0.1 and complete lysis occurred within 3.5 h, while MOIs of 0.01 and 0.001 showed complete lysis after 4.5 h (*p* < 0.005; Figures 2D–F). Determining the mean differences between all three MOIs and control was done by the two-way ANOVA test, which showed that they are significant (*p* < 0.0001).

The *in vitro* challenge tests established that the phages could be used to inactivate the MDR pathogenic strains of *Shigella* and, therefore, these phages could be useful as a bio control agent. The efficacy of those phages in controlling *Shigella* infection however has to be determined by *in vivo* studies. It is worth noting that a host population may resist long phage treatment, resulting in the emergence of bacterial insensitive mutants (BIMs). To combat this issue, a cocktail of phages may be used instead of a single phage (Amarillas et al., 2017). The use of phage cocktail with more than one phage that follows different infection mechanisms may solve this problem (Yamaki et al., 2014). The analysis of host cell lysis suggests that the MOI is directly dependent on cell death. The application of a higher number of phages on cells causes destabilization of its outer membrane, resulting in cell lysis. As this type of lysis are not due to the replication of phage and its release, it is called “lysis from without” (Brown and Bidle, 2014).

### 3.5 Infectivity of *Sfin-2* and *Sfin-6*

**Figure 3:**
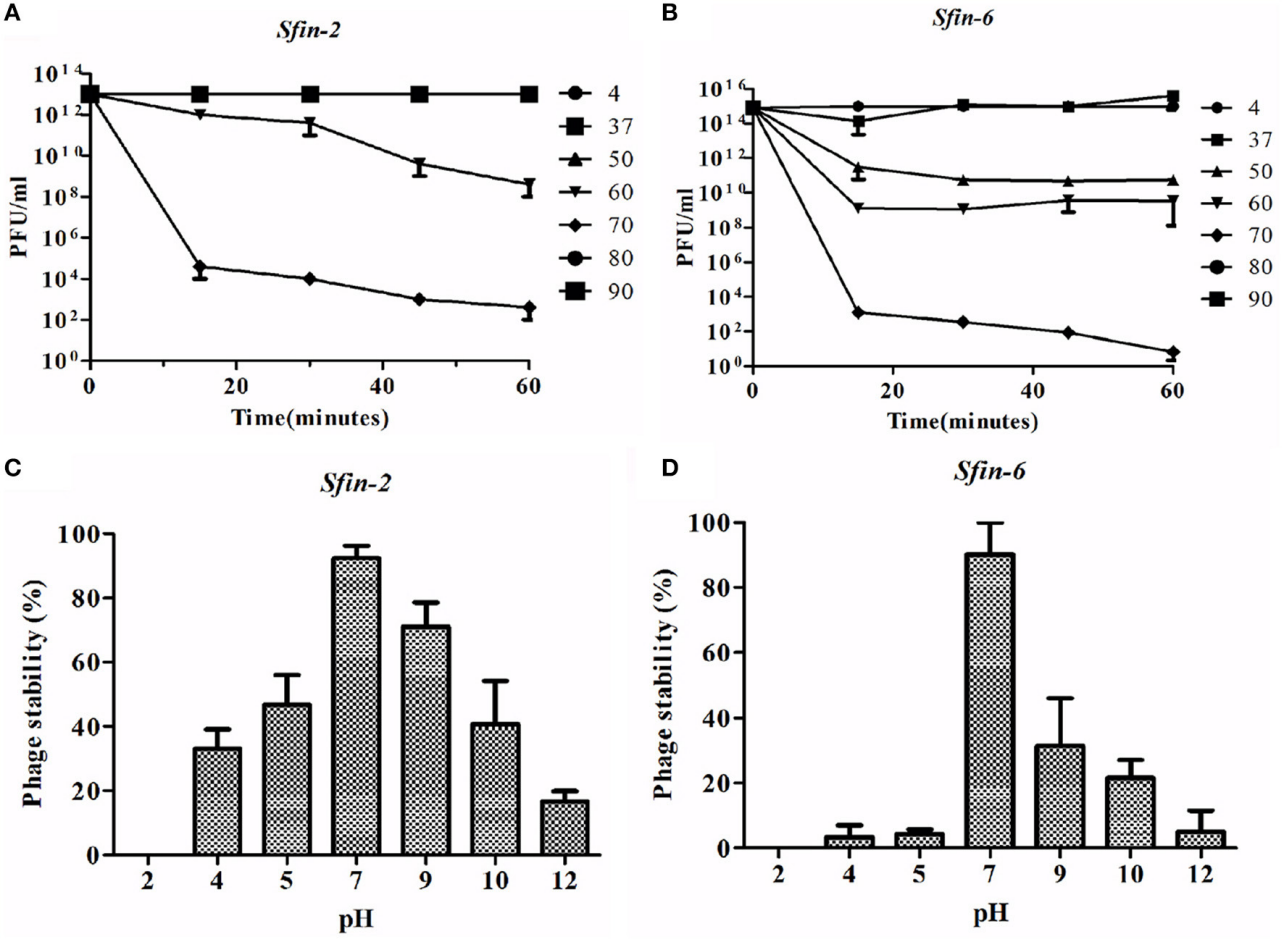
Stability of phage *Sfin-2* and *Sfin-6* in wide temperature and pH ranges. **(A, C)** Thermal stability of *Sfin-2* and *Sfin-6* phages at various temperatures. *Sfin-2* (16 × 10^13^) and *Sfin-6* (15 × 10^15^) phage particles were incubated at different temperatures in 1 ml of LB medium, and for each temperature, the number of infectious phage particles was determined using 100 μl aliquots from various time points by plaque assay against *S. flexineri* 2a. The result was plotted as mean ± SD (*n* = 3). **(B, D)** pH stability of phage *Sfin-2 Sfin-6*. In 1 ml of TM buffer having different pH, *Sfin-2* (14 × 10^9^) and *Sfin-6* (16 × 10^9^) phage particles were incubated at 37°C for 1 h, and the number of infectious phage particles from each sample was determined using 100 μl aliquots by plaque assay against *S. flexineri* 2a. The result was plotted as mean ± SD (*n* = 3).

The thermal stability test was performed to investigate the heat-resistant properties of *Sfin-2* and *Sfin-6* phages. When the *Sfin-2* phage was warmed at 37 or 50°C for 5 min, the activity remained unchanged. Then, the activity slowly decreased to 0.1–0.01% when incubated at 60 or 70°C for 5 min, and only 0.0001% activity was present when heated to 80 or 90°C for 5 min. In the case of *Sfin-6* phage, 0.01%−0.001% activity was present when incubated at 50 or 60°C for 5 min and only 0.0001% activity was retained in each case when heated at 70, 80, or 90°C for 5 min. The thermal stability of both the phages was determined by monitoring the changes of titer at different temperatures (Figures 3A, B).

The *Shigella* infection usually occurred in the intestine at acidic pH conditions (Gorden and Small, 1993). Therefore, it is essential to know the pH stability of *Sfin-2* and *Sfin-6* for controlling *Shigella* spp. For both the phages, highest activity was observed after an incubation period of 1 h at pH 7.0 at 37°C. Approximately 30% or 17% recovery of the *Sfin-2* phage and 5% or 12% recovery of the *Sfin-6* phage was found at pH 4.0 and pH 12.0, respectively (*p* < 0.005; Figures 3C, D).

Although the activity of the above phages was affected by higher and lower temperature or pH levels, remarkable activity remained at wide temperature and pH ranges. Thus, the result concluded that *Sfin-2* and *Sfin-6* phages have moderate thermal stability and a wide pH tolerance, which suggests that these phages may be used for therapeutic purposes.

### 3.6 One-step growth curve

**Figure 4:**
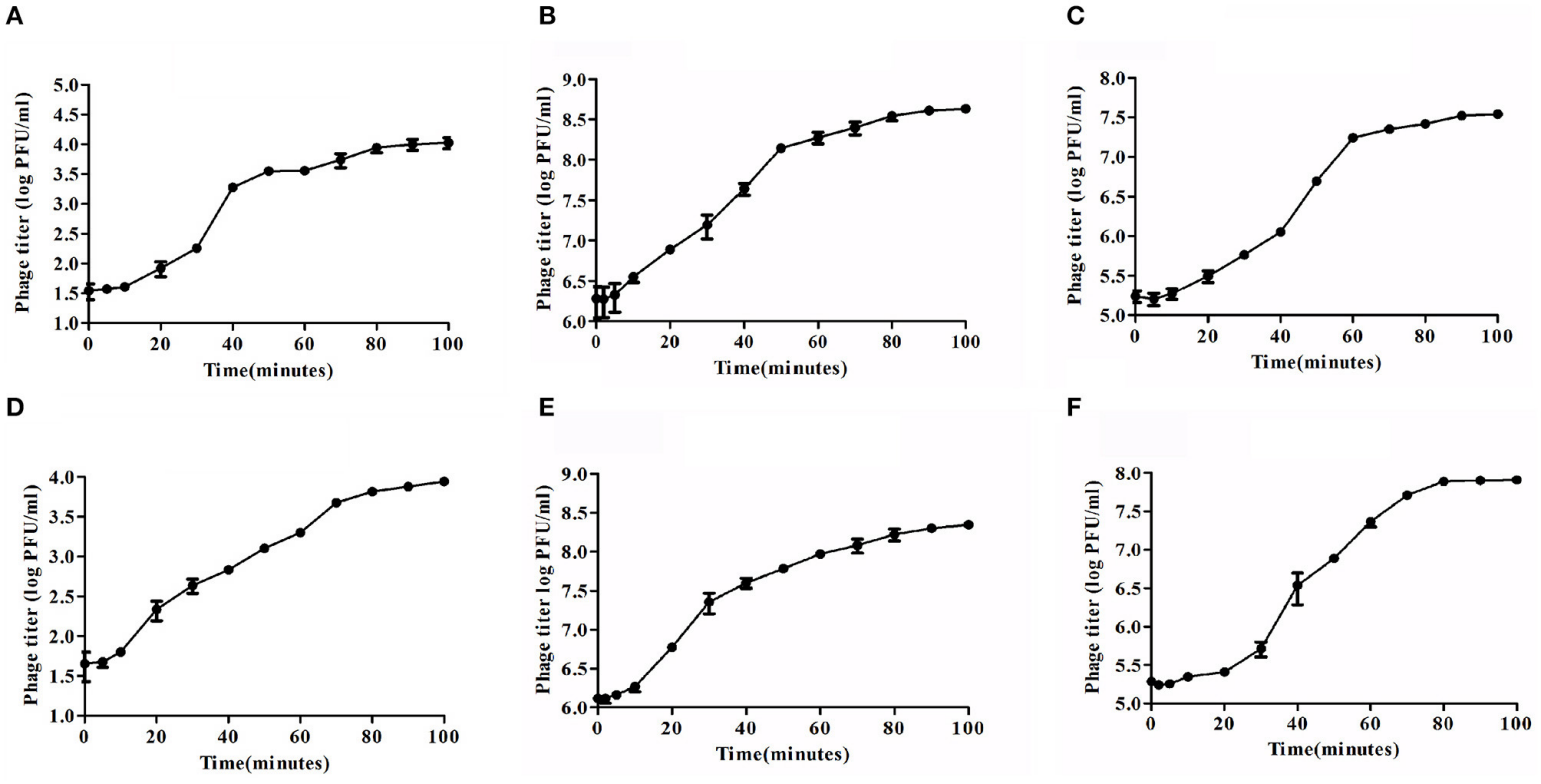
One-step growth curve of phage *Sfin-2* and *Sfin-6. Shigella flexneri* 2a, *Shigella dysenteriae* 1, and *Shigella sonnei* 1 were infected at an MOI of 0.01 at 37°C. After phage absorption, the cultures were diluted to 10^4^-fold and incubated at 37°C, and the titers in PFU per ml from the infected cultures at different time points were determined. The result was plotted as mean ± SD (*n* = 3). **(A, D), (B, E)**, and **(C, F)** Present one-step growth curves of *Sfin-2* and *Sfin-6* in *S. flexneri* 2a, *S. dysenteriae* 1, and *S. sonnei* 1, respectively.

Lytic development of *Sfin-2* and *Sfin-6* phages were investigated in one-step growth curve experiments. The adsorption above 90% for both the phages were completed within ~5–20 min. The growth curve study of *Sfin-2* phage showed a latent period of ~7 min with the average burst size of 105 PFU/cell against *S. flexneri* 2a. In the case of *S. dysenteriae* 1 and *S. sonnei* 1, latent periods were ~5 and 10 min with the average burst size of 74 and 101 PFU/cell, respectively (Figures 4A–C). *Sfin-6* exhibited a latent period of ~5 and 13 min with the average burst size of 71 PFU/cell and 163 PFU/cell for *S. flexneri* 2a and *S. dysenteriae* 1, respectively, whereas against *S. sonnei* 1, *Sfin-6* exhibited a latent period of ~13 min with the average burst size of 265 PFU/cell (Figures 4D–F).

### 3.7 Whole genome sequencing and synteny study of *Sfin-2* and *Sfin-6* phages

**Figure 5:**
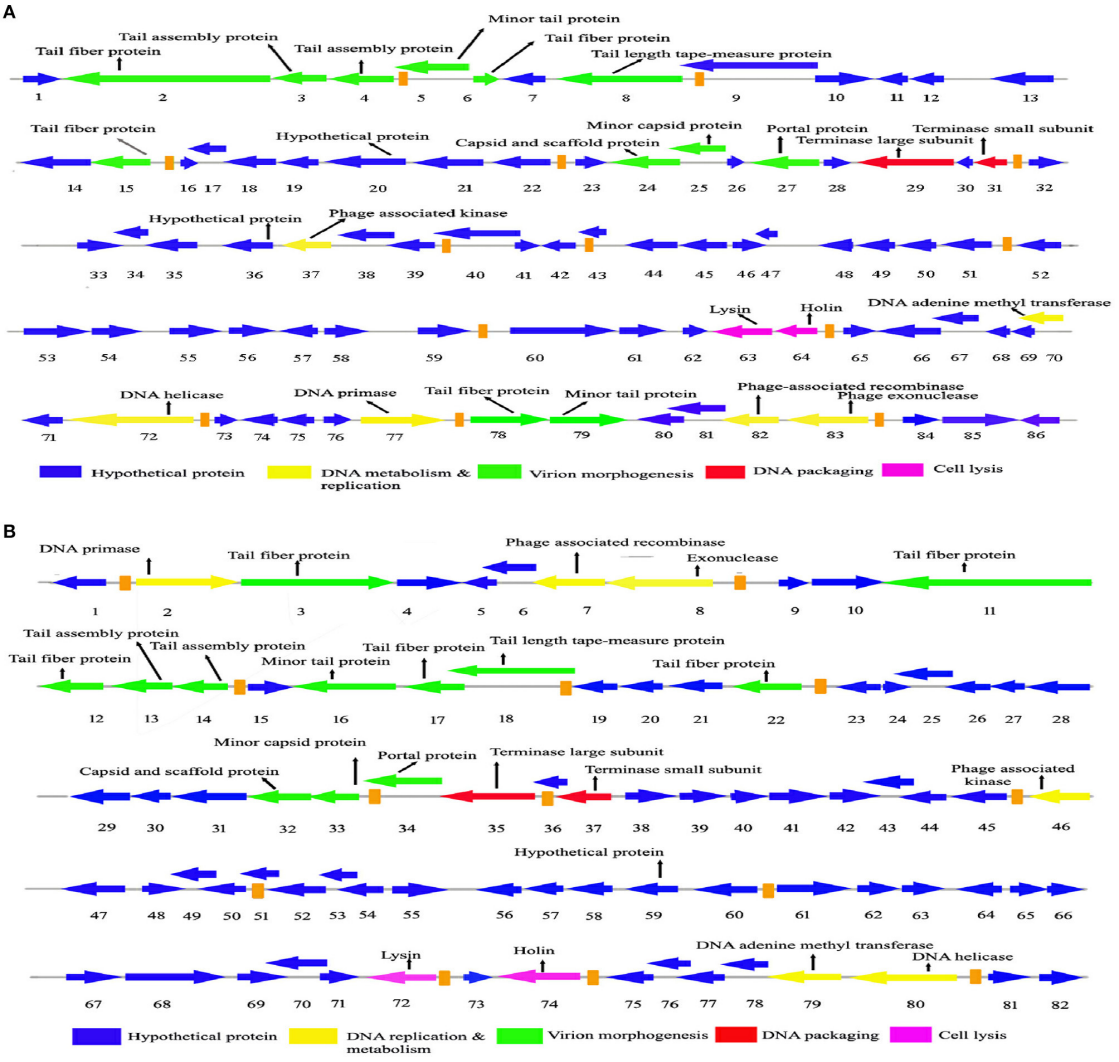
Genome organization and comparative genome analysis of *Sfin-2* and *Sfin-6*. The *Sfin-2* **(A)** and *Sfin-2* **(B)** genome maps were schematically presented. The arrows indicate the predicted CDSs and the orientation of the transcription. Predicted molecular functions of CDS were indicated by different colors: Virion morphogenesis (green arrows), DNA metabolism and replication (red arrows), DNA packaging (violet arrows), cell lysis (gray arrows), and hypothetical proteins (blue arrows).

The genome sequencing is essential to understand the phage biology. The genome of *Sfin-2* has 50,390 bp (GenBank accession number: MK972831) with 44.9% GC content. Among the 85 CDSs, 22 are rightward in orientation while others are leftward (Figure 5A) and 25 CDSs had annotated functions. The putative origin of replication and terminus location is ~201 nt and 43,001 nt, respectively, which could be predicted from the GC-skew analysis (Supplementary Figure S1A). The genome of *Sfin-6* also possesses a circular genome of 50,523 bp (GenBank accession number: MN393473) with a GC content of 48.3%. Out of the 83 CDSs, 16 are rightward in orientation, while others are leftward (Figure 5B). Among them, 23 have annotated functions. The GC skew analysis suggested that the putative origin of replication and the terminus location of phage *Sfin-6* is ~7,001 nt and 49,501 nt, respectively (Supplementary Figure S1B). No tRNA was found in both the genomes.

The whole genome BLAST analysis of *Sfin-2* and *Sfin-6* against the NCBI database showed that they are related to two phages, i.e., pSf-2 (GenBank accession number: KP085586) and phi2457T (GenBank accession number: MH917278). The genome of the *Sfin-2* phage showed 91.89% similarity with psf-2 and 98.8% similarity with phi2457T, while the genome of the *Sfin-6* phage showed 92.16% similarity with psf-2 and 99% similarity with phi2457T. The Mauve alignment of *Sfin-2, Sfin-6*, phi2457T, and pSf-2 resulted in one large LCB of 29,977 bp (green) and three small LCB of 5,338 bp (blue), 8,492 bp (red), and 6,478 bp (fluorescent green) indicating DNA regions that are homologous among the genomes. The gaps in the graphs indicate the non-identical region of the genome. Furthermore, the alignment of these phages showed some highly homologous regions with major rearrangements, which indicates that the phages share a common genome organization with different positions of genes (Supplementary Figure S2).

### 3.8 Module analysis

The comparative genome study of the two phages showed that genome sequence, genome size, GC contents, number of transcription terminator sequences, and CDSs are close to each other. Although gene sequences of predicted structural and functional proteins share high degree of homology, they are differently arranged and sometimes oppositely oriented. Maximum differences are present in the hypothetical proteins that are yet to be characterized. Approximately 72–75% genes of *Sfin-2* and *Sfin-6* are of unknown functions, and most of them have >78%−80% homology with their counterparts in pSf-2 and phi2457T genomes. The high degree of similarity among these phages may be due to complex evolutionary relationship, though they have been isolated from different geographical locations.

After annotation, the *Sfin-2* and *Sfin-6* proteins can be categorized into following functional groups: DNA metabolism and replication proteins; the downstream gene of *Sfin-2* mostly contains DNA metabolism and replication proteins, which includes 3′-phosphatase, 5′-polynucleotidekinase/CDS33, phage-associated N-6-DNA adenine-methyl transferase/CDS73, DNA helicase/CDS75, 76, DNA primase/CDS78, phage-associated recombinase/CDS82, and phage exonuclease/CDS83, while the upstream and downstream parts of the *Sfin-6* genome contains all of these proteins. The 3′-phosphatase, 5′-polynucleotide kinase belongs to the family pfam03767 that includes the C-terminal domain of the bifunctional enzyme T4 polynucleotide kinase/phosphatase PNKP. The role of The PNKP phosphatase domain is to catalyze the elimination of the 3′-phosphoryl group of DNA, RNA, and deoxynucleoside 3′-monophosphates. The enzyme N-6-DNA adenine-methyl transferase (DAM) is a member of pfam05869 which methylates GATC sequence of its own DNA to protect it from exonuclease. The counterpart of this enzyme is present in the Escherichia phage ADB-2, which shares 99% identity with *Sfin-2* and *Sfin-6*. Both the phages have helicase coding genes that belong to the pfam04851 and are involved in ATP-dependent RNA or DNA unwinding. The primase encoded by phages belongs to pfam08273. The zinc finger motif and ATP binding region of the primase/helicase at N-terminal and C-terminal, respectively, have the origin recognition property. The ERF superfamily's pfam 04404 has the phage-associated recombinase domain that contains several single-stranded annealing proteins (SSAPs) such as Red-beta, Rad 52, ERF, and RecT, which may function as Rec-A dependent and independent DNA recombination pathways. This type of recombinase encoded by the phages promotes horizontal gene transfer by homologous recombination to accelerate the evolution by intra-phage gene shuffling. The recombinase in association with phage exonuclease takes part in the replication process from fork to nucleotide metabolism. The exonuclease encoding gene of both phages encodes an exonuclease VIII that is related to pfam12684 of the PDDEXK superfamily. Thus, 3′-phosphatase, 5′-polynucleotide kinase, phage recombinase, exonuclease are involved in DNA metabolism and recombination process of the phage genome after entering the host cells.

The sequence-based prediction of the *Sfin-2* phage showed that upstream cluster genes are involved in viral head morphogenesis and tail component formation while upstream and downstream cluster genes of *Sfin-6* are involved in viral head morphogenesis and tail component formation. CDS21 of *Sfin-2* and CDS25, CDS26 of *Sfin-*6 are likely to produce phage capsid and scaffold protein belonging to Phage Mu protein F-like family which are required for viral head morphogenesis. Head and tail junction proteins, known as portal proteins, allow the phage genome into the pro head as a part of the packaging motor (Lokareddy et al., 2017). CDS23 and CDS24 of *Sfin-2* and CDS28 and CDS29 of *Sfin-*6 encode phage large and small terminase subunits, which are involved in the packaging of concatameric DNA in phage capsids (Mobberley et al., 2008). CDS1, CDS4, CDS5, CDS10, CDS78, and CDS79 probably encode the tail component for *Sfin-2*, whereas CDS2 and CDS3 direct the synthesis of the protein responsible for tail assembly. CDS2, CDS8, CDS11, CDS12, and CDS16 encode the tail component for *Sfin-6*, whereas CDS9 and CDS10 direct the synthesis of the protein responsible for tail assembly. CDS6 and CDS7 for *Sfin-2* and CDS13 for *Sfin-6* encode tail tape measure protein which are the second largest genes of the phage genome. The tail length of the lambdoid phages may be hypothetically determined by the total amino acid residue of tail tape measure protein where a single amino acid is corresponding to ~0.15 nm (Katsura, 1990). According to this hypothesis, the probable tail lengths of *Sfin-2* and *Sfin-6* phages are 140 and 138 nm long, respectively, which are much closed to the measured length of 145 and 148 nm, respectively.

CDS22 and CDS23 of *Sfin-2* and CDS28 and CDS29 of *Sfin-6* encode the large and small terminase subunit, respectively. These are mainly involved in ATP-dependent DNA packaging system.

CDS64 of *Sfin-2* and CDS71 of *Sfin-6* encode cell lysis protein lysin while CDS65 for *Sfin-2* and CDS72 for *Sfin-6* encode holins, which play an important role in host cell destruction during the burst step of the phage life cycle. After the assembly of new progeny of phages, the host cell lysed by a dual lysis system followed by a pore-forming holin protein and a cell wall degrading enzyme known as phage lysozyme or endolysin. Both lysin and holin encoding genes are located contagiously at the terminal part of *Sfin-2* and *Sfin-6* genomes. The lysin-coding gene encodes 162 amino acids along with phage lysozyme/endolysin belonging to the pfam00959 family found in dsDNA phages. Holin in association with other members of pfam 00959 cleaves the ß1,4-glycosidic linkage of polysaccharide present in the bacterial membrane (Ziedaite et al., 2005). CDS76 of *Sfin-2* and CDS83 of *Sfin-6* encode transcriptional regulatory cro protein that belongs to the HTH_XRE superfamily. Phages may use this protein to regulate transcriptional timing in the gene expression. Therefore, the presence of lysis genes and the absence of lysogeny-related genes in both the genomes clearly indicate that the phages are potent lytic phages.

### 3.9 Determination of genome ends

Whole genome sequencing followed by the assembly of both phages revealed that they had a double-stranded DNA genome. In tailed bacteriophages, a linear genome is expected within the channel of the portal protein where only one dsDNA can pass. Therefore, the head contains a linear genome with different types of ends. However, PCR with the primers designed at the two ends of the whole genome sequence confirmed the circular nature of the *Sfin-2* and *Sfin-6* phage genomes (Supplementary Table S1, Supplementary Figure S3). Consequently, two PCRs at the adjacent of the 5′ and 3′ end of the genome were taken as the positive control (data not shown).

**Figure 6:**
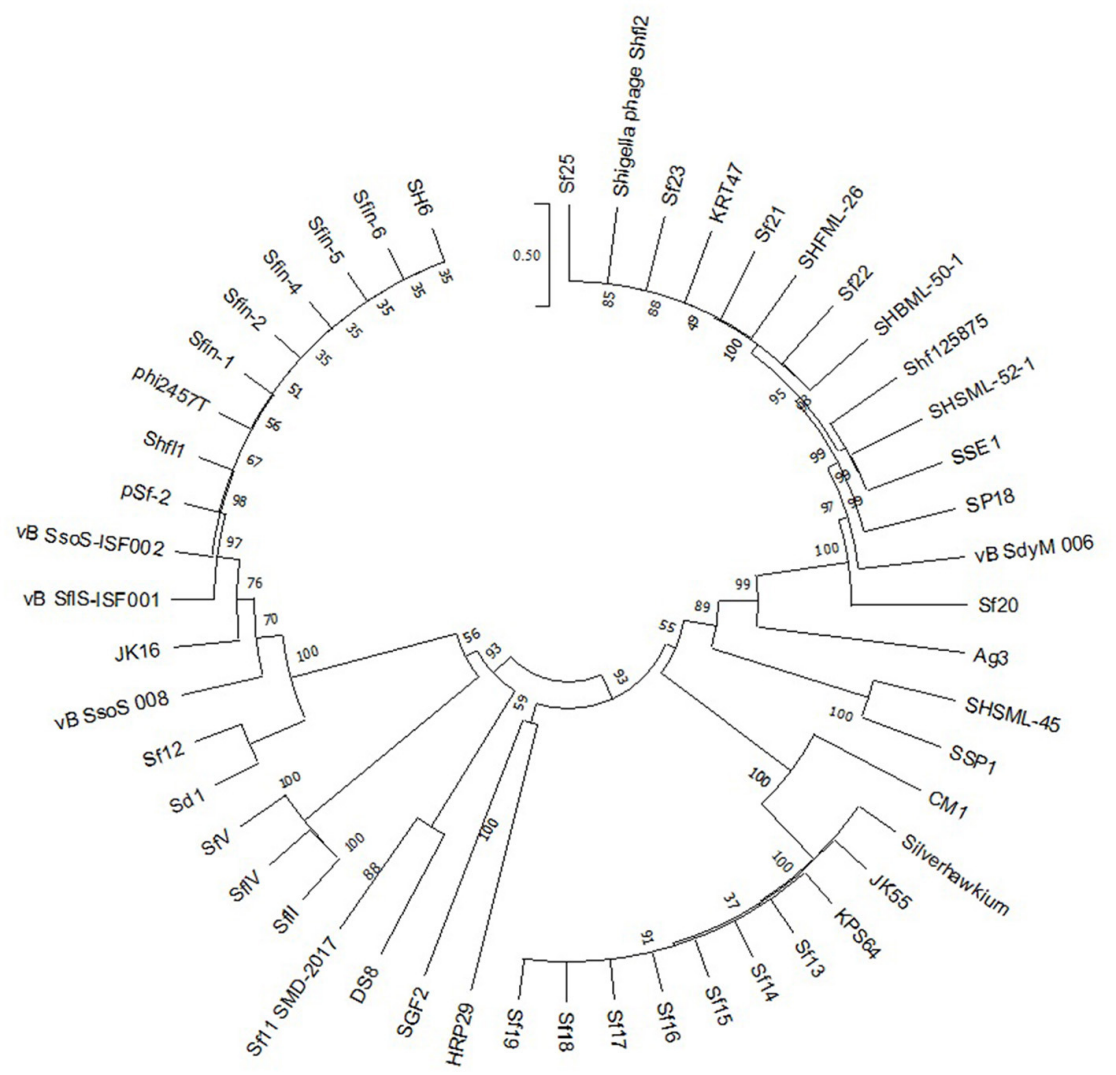
Phylogenetic study of *Sfin-2* and *Sfin-6* phages with related phages. The phylogenetic analysis based on the large terminase subunit of known packaging mechanisms phages. The bootstrap analysis was performed with 1,000 repetitions. The terminase large subunits were compared in the MEGA 7.0 version using the neighbor-joining method.

Phage terminase is one of the most conserved protein that creates the virion end, and this enzyme is one of the most conserved phage proteins within the group. Therefore, the comparative analysis of terminase amino acid sequence of a phage results in the same clusters with others that generate similar ends. According to the phylogenetic analysis of the large terminase subunit, *Sfin-2* and *Sfin-6* clustered with the terminase of *Shigella* phages ISF002, Shfl1, psf-2, ISF001, and *E. coli* phage ADB-2 which belong to T1 family of phage (Figure 6). According to the cluster, it is predicted that both the genomes have direct terminal repeats with possible circular permutation. In such a circularly permutated headful packaging phage category, the site of initiation cleavage is not precise and several initiation cuts are spread on concatamers. Thus, for this reason, the chromosome length of individual virions are not precise. The abovementioned types of phages are expected to contain all the fragments of the restriction digestion of the circular phage genome as well as of undigested phage DNA along with submolarpac fragment-like P22 genome (Casjens et al., 2004). The pac fragments, such as phage sf6 and ES18, may not be detected for imprecise series initiation cleavage. Hence, as a result, a blur background will be observed due to variable lengths of terminal fragments.

**Figure 7:**
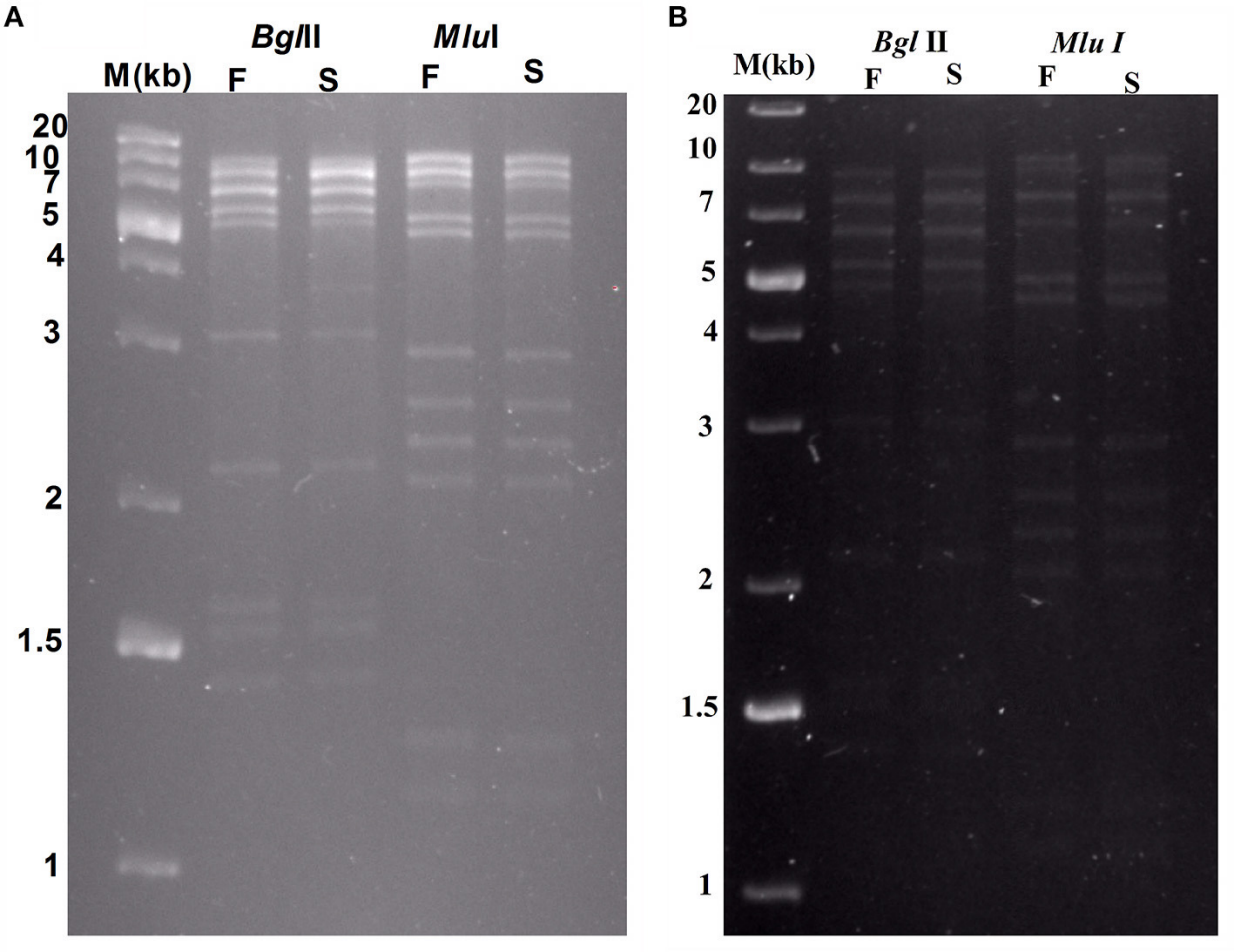
Enzymatic analysis of *Sfin-2* and *Sfin-6* genomic DNA. Phage *Sfin-2* **(A)** and *Sfin-6* **(B)** DNA was completely digested with *Bgl*II and *Mlu*I and the products were analyzed by 0.8% agarose gel electrophoresis. Lane M indicates the 1 kb Plus DNA Ladder. Lanes F and S indicate that the digests were heated to 80°C for 15 min and then cooled fast on ice or slow at room temperature, respectively.

Restriction digests of *Sfin-2* and *Sfin-6* phage genomes by *Bgl*II and *Mlu*I were warmed at 80°C and then cooled down slowly or rapidly, and no difference was noticed between slow- and fast-cooled sets for both the enzymes. However, instead, longer fragments were observed which indicated the absence of cohesive ends in both the phage genomes. Additionally, blur background was also observed in electrophoresis gel. For the phages that contain cohesive ends are expected to anneal and appear as a longer fragment in gel electrophoresis. This result indicates that both *Sfin-2* and *Sfin-6* phages are the T1-like headful packaging phage (Figure 7).

### 3.10 Characterization of the host receptor

The important aspect of phage infection is the identification of host cell surface receptor for adsorption. The nature and location of the host cell receptors vary greatly depending on the phage and host (Stone et al., 2019). They range from peptide sequences to polysaccharide moieties. In fact, bacterial capsules or slime layer appendages may also act as the receptor of the phages (Sorensen et al., 2011; Bae and Cho, 2013; Mahony and van Sinderen, 2015; Dowah and Clokie, 2018; Ha et al., 2019; Leprince and Mahillon, 2023).

**Figure 8:**
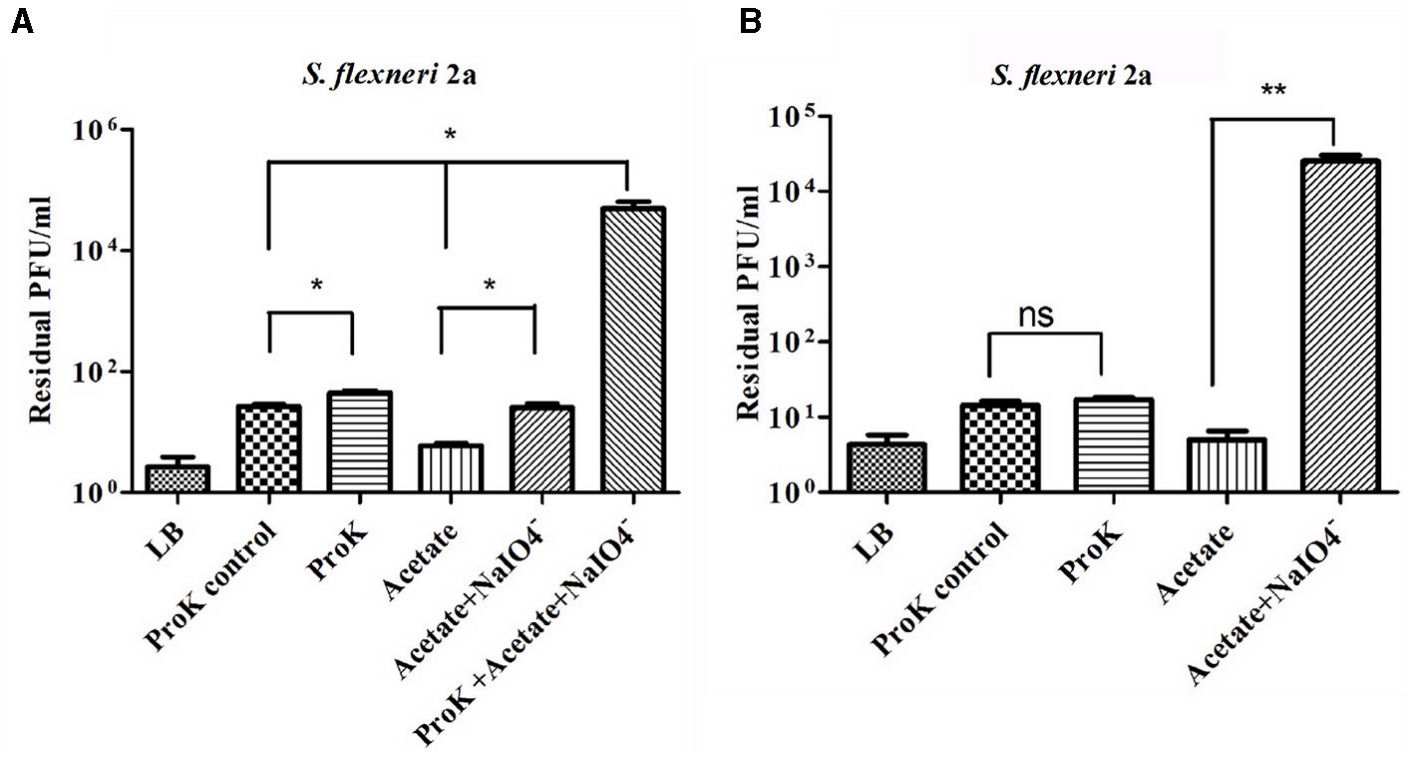
*Sfin-2* and *Sfin*-*6* infections on proteinase K and periodate-treated host. The effect of proteinase K and sodium periodate with proteinase K on *Sfin-2* **(A**). The effect of sodium periodate and sodium periodate with proteinase K on *Sfin-6* **(B)**. *Shigella flexneri* 2a culture (OD600 = 0.3 U) was treated with proteinase K (250 mg/ml), sodium periodate (200 mM ${\text{NaIO}}_{4}^{-}$), and sodium periodate with proteinase K followed by infection at an MOI of 0.0001. Upon centrifugation, the phage titer in the supernatant was determined by plaque assay. Cells suspended in LB medium, cells incubated at 55°C in LB medium, and cells in acetate buffer were used as control. The results are shown as residual PFU percentages. The phage titer in the control supernatant was set to 100%. The mean ± SD of three independent experiments is indicated. To determine the significance of the differences between group means, unpaired *t*-tests were performed between the controls and the tests. Asterisks indicate the significance levels (ns, *p* > 0.05; *p ≤ 0.05; **p ≤ 0.005).

*Shigella* spp. belong to gram-negative bacteria and exhibit complex LPS and protein in their outer membrane structures. So, either LPS or protein or both of them may involve in phage host interaction (Cohen et al., 2019; Qasim et al., 2022). Therefore, it is very much essential to identify the actual component which serves as the receptor of the phages. Based on the strategy of Kiljunen et al., the outer membrane LPS and protein of *S. flexneri* 2a were degraded by periodate and proteinase K before the infection (Kiljunen et al., 2011; Stone et al., 2019). The *Sfin-2* phage showed no changes in infection efficiency with or without proteinase K and periodate-treated host. In contrast, a high number of phage particles remained unabsorbed when hosts were pre-treated with proteinase K and periodates at a time. Thus, this experiment suggests that the adsorption of phage *Sfin-2* phage to the host is mediated either by the outer membrane of the protein or complex LPS structure (Figure 8A). In the case of *Sfin-6* phage, a high number of residual phage were present when *S. flexneri* 2a cells were pre-treated with periodates whereas no significant change in efficacy of infection was observed when the host cell was pre-treated with proteinase K. Therefore, this result suggests that the adsorption of *Sfin-6* phage to the host is mediated by the outer membrane LPS structure but not the protein (Figure 8B).

### 3.11 Inactivation of *S. flexneri* 2a cells with *Sfin-2* and *Sfin-6* by singly or cocktail of two phages in raw chicken sample

**Figure 9:**
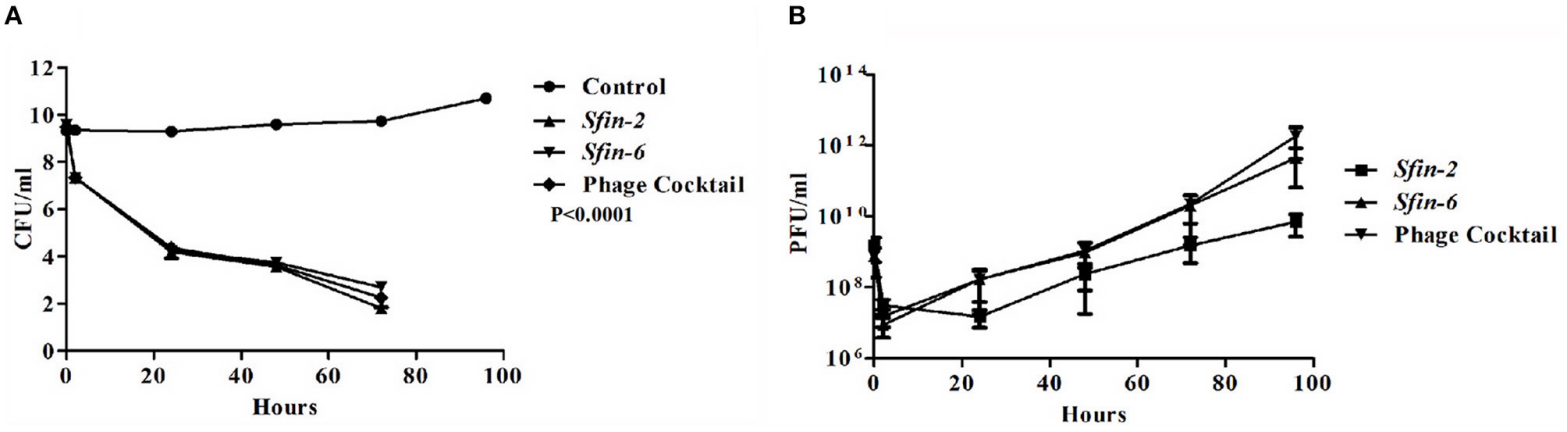
Inactivation of *Shigella flexneri* 2a by the single phage or cocktail of phages on chicken. Mid-log phase culture of *S. flexneri* 2a was inoculated on the surface of the chicken and allow for incubation of 10 min. Afterward, a single phage or cocktail form of phages was added (MOI of 0.1) and kept at 4°C up to 96 h. As the negative control, chicken pieces were inoculated with *S. flexneri* 2a without any phages. At different time intervals, the viability of *S. flexneri* 2a was determined by the spread plate method, and the number of phages was measured by plaque assay. **(A)** Reduction in viable count of cells showed after 48 h of incubation. A two-way ANOVA test indicated significant difference between control and phage infected sets (*p* < 0.0001, *n* = 3). **(B)** The number of phages decreased initially for 2 h but after that, the number increased gradually.

Foodborne infections are major threats to food safety in the present times. Recently, nearly two billion individuals are suffering from foodborne illnesses, resulting in 1 million deaths around the world (Kirk et al., 2015). Traditional food sanitation techniques can be effective in reducing the presence of pathogens in food with varying degrees. However, these methods have plenty of disadvantages, including the damage of organoleptic qualities of foods, and most importantly, chemicals used in food safety eliminate “good” microbes that are beneficial in the natural preservation of foods (Moye et al., 2018). Therefore, it is preferable to use bacteriophages as an alternative tool to combat the problems, as the bacteriophages are host-specific and kill their respective hosts without changing organoleptic properties of foods with low-cost large scale production, self-replicating nature, and low toxicity (Loc-Carrillo and Abedon, 2011; Perera et al., 2015). The use of bacteriophages to control MDR pathogens is gaining more interest in recent times (Rogovski et al., 2021). Zhang et al. (2013) reduced the *Shigella* load on ready-to-eat spiced chicken by at least 2log_10_ after using *Shigella*-specific phages. Shahin et al. (2018) reported significant reduction of *Shigella* contamination in food items after the uses of *Shigella-*specific phages (Shahin and Bouzari, 2018). In this study, the two polyvalent *Shigella* phages, *Sfin-2* and *Sfin-6*, were used either individually or in a cocktail form to reduce the *Shigella* load on raw chicken samples. The result showed significant differences in the number of viable bacterial cells between the control and single phage or cocktail-treated chicken sample. No *Shigella* cells were found in control. The concentration of viable bacterial cells on the treated chicken sample by both single and cocktail of phages decreased by ~2log_10_ of the initial count. The major reduction in cell concentration occurred after 48 h of incubation, and almost complete lysis occurred after 72 h. At 96 h of incubation, the viability of cells reduced below the level of detection (Figure 9A).

The number of active phages were also measured at each time point after treatment. The number of phage decreased by ~2log_10_ of the initial value, 2 h after the addition of single or cocktail phages. Afterward, the number gradually increased with time in both single and cocktail of phages (Figure 9B).

## 4 Conclusion

Shigellosis is still one of the major threats in developing countries, and multidrug resistance of *Shigella* spp. has made the situation even worse. Therefore, to combat the situation, phages are gaining more popularity as an alternative therapeutic agent to resist pathogenic bacterial infection. Other than that, phages are also useful to treat foods infected with MDR bacterial pathogens. In the present study, we have characterized two novel thermostable and wide pH-tolerant *Siphoviridae* phages, *Sfin-2* and *Sfin-6*, that have specificity and lytic properties against important enteropathogenic MDR *Shigella* spp. The article represents the complete physical as well as genomic characterizations of the *Sfin-2* and *Sfin-6* phages that include sequence analysis, genome annotations, and differences between gene rearrangements among the other closely related phages. Genome analysis is very crucial for the study and use of phages to regulate host bacterial machinery. Phylogenetic analysis confirms that *Sfin-2* and *Sfin-6* belong to the T1-like phage family, which may be packaged by the headful packaging method. The phage–host interaction study through specific receptor molecules suggested that the phage *Sfin-2* can interact with both LPS-O antigen and protein, while *Sfin-6* only interacts with the LPS-O antigen of the outer cell membrane of the host cells. Further studies of the activity of *Sfin-2* and *Sfin-6* phages on *Shigella-*infected raw chicken meat either in a single or cocktail form ensure that both the phages have the potential to reduce the number of MDR *Shigella* load from the meat samples.

From the present study, it can be concluded that the *Sfin-2* and *Sfin-6* phages can be satisfactory therapeutic agents either in a single or cocktail form, and further studies on these two phages will be helpful to apply it for the treatment of shigellosis as well as for the preservation of meat.

## Data availability statement

The datasets presented in this study can be found in online repositories. The names of the repository/repositories and accession number(s) can be found in the article/Supplementary material.

## Author contributions

SA, SR, CG, and NG conceived and designed the entire study, performed the experiments, analyzed the results, and prepared the manuscript. DM and VB supplied the clinical samples. SA, NG, and SD analyzed the phage structure. RJ helped in genome analysis. All authors wrote, read, and approved the final manuscript.

## Supplementary material

Supplementary Figure S1 GC-skew plot. The cumulative graph displays the global minimum and maximum. The window size of 1,000 bp and a step size of 100 bp were used to calculate the global minimum and maximum. The blue and red lines represent the GC-skew and the cumulative GC-skew, respectively. **(A, B)** The putative origin of replication (201 nt) and the putative terminus location (43,001 nt) of *Sfin-2* and putative origin of replication (7,001 nt) and the putative terminus location (4,9501 nt) of *Sfin-6* can be predicted from the minimum and maximum of a GC-skew plot.

Supplementary Figure S2 Genome comparison of phage *Sfin-2* and *Sfin-6* with closely related phage spSf-2 and phi2457T were constructed using the Mauve progressive alignments. Boxes with identical colors represent LCBs. White rectangles represent the CDSs. Rectangles below the horizontal line indicate opposite orientations. The graph below the horizontal line indicates relative inverted genome segments.

Supplementary Figure S3 PCR primers were designed to confirm the nature of the genome end of *Sfin-2* **(A)** and *Sfin-6* **(B)**. At the 5′ (1A, 1B for *Sfin-2* and 3A, 3B for *Sfin-6*) and 3′ (2A, 2B for *Sfin-2* and 4A, 4B for *Sfin-6*) end primers were designed for positive control. Same primers from 5′ end (1B for *Sfin-2*, 3B for *Sfin-6*) and from 3′ end (2A for *Sfin-2*, 4A for *Sfin-6*) were used for PCR to charecterize the circular or linear nature of phage DNA. As per design, 410 bp PCR product would be expected for *Sfin-2*, 328 bp for *Sfin-6* if the genomes are circular **(C)** PCR products were run in 1.5% agarose gel. The lane M indicates the 100 bp DNA Ladder. 1B and 2A lane and 3B and 4A lane indicate the PCR product using 1B, 2A, and 3B, 4A primers for *Sfin-2* and *Sfin-6* respectively. Control lane indicates the non-template PCR control by using the same primers for each phage separately.

Supplementary Table S1 List of primers used for PCR of the genome ends *Sfin-2* and *Sfin-6*.
