# Supplementary material for: Characterizations of novel broad-spectrum lytic bacteriophages Sfin-2 and Sfin-6 infecting MDR Shigella spp. with their application on raw chicken to reduce the Shigella load
Source: Front Microbiol. 2023 Nov 29;14:1240570. doi: 10.3389/fmicb.2023.1240570 (PMC10716491; doi:10.3389/fmicb.2023.1240570)
Supplement: Supplementary Figure S1 — GC-skew plot. The cumulative graph displays the global minimum and maximum. The window size of 1,000 bp and a step size of 100 bp were used to calculate the global minimum and maximum. The blue and red lines represent the GC-skew and the cumulative GC-skew, respectively. (A, B) The putative origin of replication (201 nt) and the putative terminus location (43,001 nt) of Sfin-2 and putative origin of replication (7,001 nt) and the putative terminus location (4,9501 nt) of Sfin-6 can be predicted from the minimum and maximum of a GC-skew plot. [file Data_Sheet_1.zip › 1240570_Giri_Table_3.docx]

| **Name** | **Primer sequence** | **Position on genome** |
| --- | --- | --- |
| 1. ***Sfin-2* 5' fwd (1A)** | 5’ GGCGGGACTATGGCGATAAA 3’ | **79 nt-98 nt (5’ end)** |
| 1. ***Sfin-2* 5' rev**   **(1B)** | **5’ ACACACGCCCTTGTATCGAA 3’** | **182 nt-201 nt (5’ end)** |
| 1. ***Sfin-2* 3' fwd**   **(2A)** | **5' ATGAGCGCTGCATTTGTTGG 3’** | **50181 nt- 50200 nt**  **(3’ end)** |
| 1. ***Sfin-2* 3' rev**   **(2B)** | 5' TAGTTTGCATCCCTGCACCA 3’ | **50304 nt- 50323 nt**  **(3’ end)** |
| 1. ***Sfin-6* 5' fwd**   **(3A)** | 5' AGAAAGAGGACGTCTTGCCG 3’ | **40 nt- 50 nt (5’ end)** |
| 1. ***Sfin-6* 5' rev**   **(3B)** | 5' TTTACCTCCACAGTTGGGGC 3’ | **142 nt- 162 nt (5’ end)** |
| 1. ***Sfin-6* 3' fwd**   **(4A)** | 5'TTCGATCTGAAGTTTGATGGGAA 3’ | **50234 nt- 50256 nt (3’ end)** |
| 1. ***Sfin-6* 3' rev**   **(4B)** | 5'CCAAATAAAAAACGCGTAGAAGGA 3’ | **50377 nt- 50400 nt (3’ end)** |
