# Supplementary figures and images for: Characterizations of novel broad-spectrum lytic bacteriophages Sfin-2 and Sfin-6 infecting MDR Shigella spp. with their application on raw chicken to reduce the Shigella load
Source: Front Microbiol. 2023 Nov 29;14:1240570. doi: 10.3389/fmicb.2023.1240570 (PMC10716491; doi:10.3389/fmicb.2023.1240570)

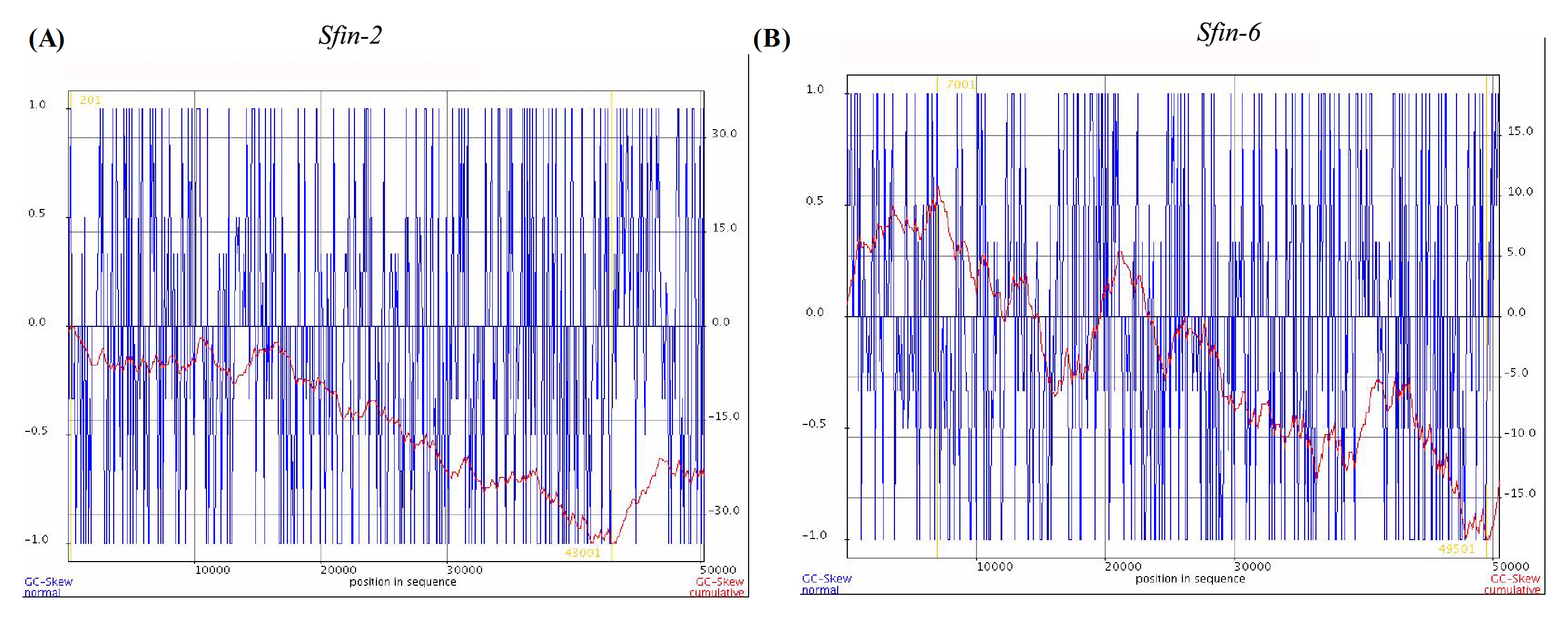

Supplement: Supplementary Figure S1 — GC-skew plot. The cumulative graph displays the global minimum and maximum. The window size of 1,000 bp and a step size of 100 bp were used to calculate the global minimum and maximum. The blue and red lines represent the GC-skew and the cumulative GC-skew, respectively. (A, B) The putative origin of replication (201 nt) and the putative terminus location (43,001 nt) of Sfin-2 and putative origin of replication (7,001 nt) and the putative terminus location (4,9501 nt) of Sfin-6 can be predicted from the minimum and maximum of a GC-skew plot. [file Data_Sheet_1.zip › 1240570_Giri_Image 1.jpg]

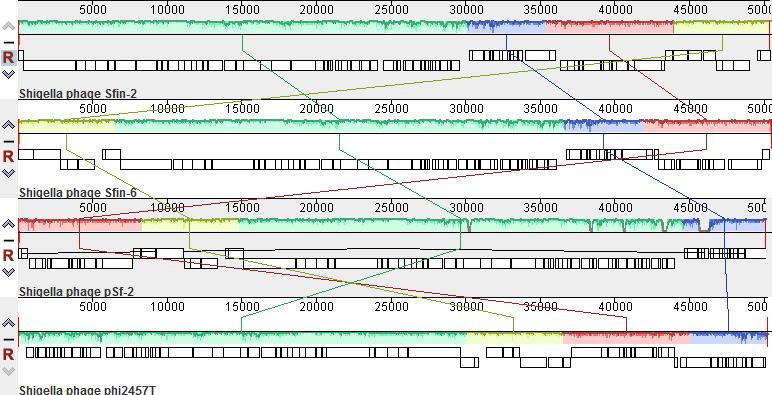

Supplement: Supplementary Figure S1 — GC-skew plot. The cumulative graph displays the global minimum and maximum. The window size of 1,000 bp and a step size of 100 bp were used to calculate the global minimum and maximum. The blue and red lines represent the GC-skew and the cumulative GC-skew, respectively. (A, B) The putative origin of replication (201 nt) and the putative terminus location (43,001 nt) of Sfin-2 and putative origin of replication (7,001 nt) and the putative terminus location (4,9501 nt) of Sfin-6 can be predicted from the minimum and maximum of a GC-skew plot. [file Data_Sheet_1.zip › 1240570_Giri_Image_2.jpg]

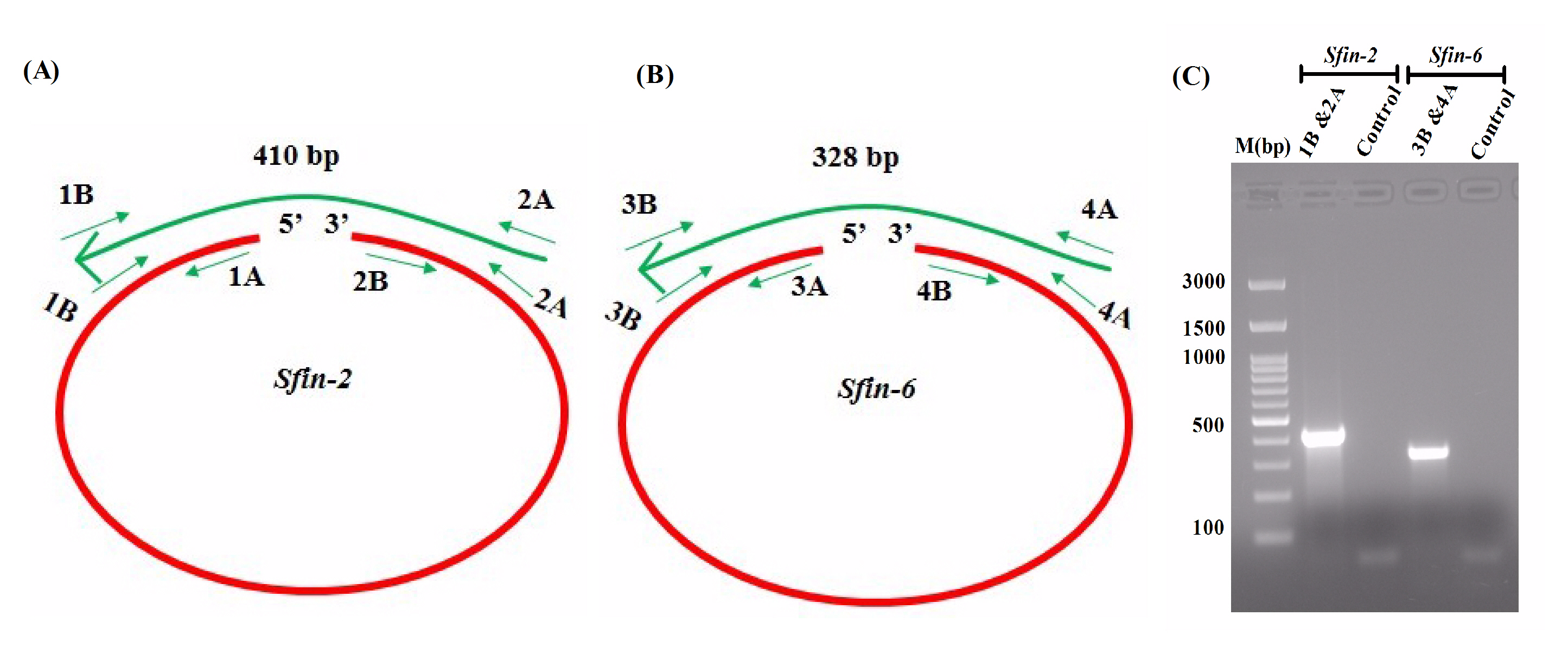

Supplement: Supplementary Figure S1 — GC-skew plot. The cumulative graph displays the global minimum and maximum. The window size of 1,000 bp and a step size of 100 bp were used to calculate the global minimum and maximum. The blue and red lines represent the GC-skew and the cumulative GC-skew, respectively. (A, B) The putative origin of replication (201 nt) and the putative terminus location (43,001 nt) of Sfin-2 and putative origin of replication (7,001 nt) and the putative terminus location (4,9501 nt) of Sfin-6 can be predicted from the minimum and maximum of a GC-skew plot. [file Data_Sheet_1.zip › 1240570_Giri_Image_3.jpg]
